# Underweight Versus Obesity and Gastrointestinal Cancer Mortality in Type 2 Diabetes: A Nationwide Cohort Study

**DOI:** 10.1002/jcsm.70408

**Published:** 2026-10-21

**Authors:** Ji‐Hong Park, Kyungdo Han, Hun Jee Choe, Se Eun Park, Won‐Young Lee, Sun‐Joon Moon, Eun‐Jung Rhee

**Affiliations:** ^1^ Department of Internal Medicine Kangbuk Samsung Hospital, Sungkyunkwan University School of Medicine Seoul Republic of Korea; ^2^ Department of Statistics and Actuarial Science Soongsil University Seoul Republic of Korea; ^3^ Division of Endocrinology and Metabolism, Department of Internal Medicine Hallym University Dongtan Sacred Heart Hospital Hwaseong Republic of Korea; ^4^ Division of Endocrinology and Metabolism, Department of Internal Medicine Kangbuk Samsung Hospital, Sungkyunkwan University School of Medicine Seoul Republic of Korea

**Keywords:** body mass index, cancer mortality, gastrointestinal cancer, type 2 diabetes mellitus, underweight

## Abstract

**Background:**

Underweight is a well‐established risk factor for cancer mortality, yet its impact relative to obesity remains unclear in individuals with type 2 diabetes mellitus (T2DM), where current guidelines focus primarily on obesity management with limited guidance on underweight‐related risks. We evaluated the association between body mass index (BMI) and gastrointestinal (GI) cancer mortality across the full BMI spectrum in individuals with T2DM.

**Methods:**

This nationwide population‐based cohort study utilized the Korean National Health Insurance Service database. We included 2 507 976 adults with T2DM screened in 2015–2016 and followed through 2022, with a 1‐year lag period applied to minimize reverse causality. BMI was categorized into eight groups: severe underweight (< 16.0 kg/m^2^), moderate underweight (16.0–16.9 kg/m^2^), mild underweight (17.0–18.4 kg/m^2^), normal weight (18.5–22.9 kg/m^2^), overweight (23.0–24.9 kg/m^2^), Class I obesity (25.0–29.9 kg/m^2^), Class II obesity (30.0–34.9 kg/m^2^) and Class III obesity (≥ 35.0 kg/m^2^). Multivariable‐adjusted hazard ratios (aHRs) for GI cancer mortality were estimated using Cox proportional hazards models adjusting for clinical variables.

**Results:**

During 5.75‐year mean follow‐up, 10 978 GI cancer deaths occurred (975 oesophageal, 4391 gastric, 5317 colorectal and 295 other GI cancers including small intestine and anal cancer). A reverse J‐shaped association was observed with mortality nadir at BMI 25.0–29.9 kg/m^2^, which served as the reference group. Underweight was consistently associated with increased mortality across all severity categories: mild underweight aHR 2.54 (95% CI 2.26–2.85), moderate underweight aHR 2.51 (95% CI 1.96–3.20) and severe underweight aHR 2.96 (95% CI 2.14–4.09), with the highest risk observed in severe underweight. Notably, all underweight categories were associated with substantially higher mortality risk than Class III obesity (aHR 1.35, 95% CI 1.05–1.73). Cancer‐specific analyses revealed that underweight was associated with the highest mortality risk for oesophageal cancer (aHR 13.23 [95% CI 6.77–25.85] for severe underweight), followed by gastric and colorectal cancers, whereas obesity significantly increased mortality only for colorectal cancer (aHR 1.52 [95% CI 1.10–2.08] for Class III obesity). Underweight was not significantly associated with GI cancer incidence after adjustment, and associations remained largely robust in 2‐, 3‐, and 4‐year lag and competing risk analyses, except for colorectal cancer.

**Conclusions:**

In individuals with T2DM, underweight status is associated with substantially higher GI cancer mortality risk than obesity, with severe underweight showing nearly threefold higher risk compared to the reference group (BMI 25.0–29.9 kg/m^2^). These findings suggest that underweight, rather than obesity alone, warrants greater clinical attention as a determinant of GI cancer mortality in this population.

## 1 Introduction

Underweight status and compromised nutritional reserves, including reduced lean body mass and depleted protein stores, are well‐established adverse prognostic factors for cancer outcomes [1]. Numerous studies have demonstrated that underweight individuals experience significantly elevated mortality risk across various cancer types [2], particularly gastrointestinal cancers [3, 4], where nutritional vulnerability is further exacerbated by impaired nutrient absorption and increased metabolic demands [5]. This poor prognosis has been attributed to diminished capacity to withstand intensive cancer treatments, highlighting the critical role of body composition and nutritional status in cancer survivorship [6].

Despite these well‐documented risks, current clinical guidelines for both cancer and metabolic diseases predominantly emphasize weight loss and obesity management, with limited attention to the dangers of low body weight or optimal body mass index (BMI) ranges [7, 8]. This focus on obesity reduction may inadvertently overlook individuals at the opposite end of the weight spectrum who face substantial mortality risk. The situation becomes particularly complex in individuals with type 2 diabetes mellitus (T2DM), where obesity has generally been linked to worse prognoses for cardiovascular and other clinical outcomes [9, 10]. Given that cancer has emerged as a leading cause of death among individuals with T2DM [11], who exhibit higher cancer incidence and mortality compared to the general population [12], understanding the differential impact of underweight versus obesity on cancer outcomes in this population has become increasingly important.

Gastrointestinal cancers represent a substantial proportion of cancer‐related mortality and are particularly prevalent in individuals with T2DM [13, 14, 15]. However, a critical gap exists in research examining how underweight compares to obesity in terms of gastrointestinal cancer mortality across the full BMI spectrum in this population. While underweight clearly poses significant risks in the general population, uncertainty remains regarding whether this relationship holds in individuals with T2DM, who experience compromised metabolic conditions where obesity has traditionally been associated with adverse outcomes. Based on these considerations, we conducted a large‐scale nationwide cohort study to comprehensively investigate the impact of BMI on gastrointestinal cancer mortality risk among individuals with T2DM. To robustly clarify the role of underweight, our analysis included categorizing underweight into distinct severity levels.

## 2 Methods

### 2.1 Korean National Health Insurance Service (NHIS) Database

The Korean NHIS is a public health insurance program that is currently covering approximately 50 million South Koreans. Enrollment in the NHIS is mandatory for all South Korean citizens, and individuals aged 20 years and older are provided with a national health examination through the NHIS. This national health examination includes a standardized questionnaire assessing medical history, current medication use, and lifestyle habits, such as alcohol consumption, smoking and physical activity. Additionally, it incorporates anthropometric measurements, including height, weight and waist circumference. The examination further includes fundamental laboratory tests, such as complete blood counts, liver and renal function tests, fasting blood glucose and lipid profiles. Serial data collected from health examinations, along with diagnostic codes based on the International Classification of Diseases, Tenth Revision, Clinical Modification (ICD‐10‐CM), are stored in the NHIS database. Moreover, these data can be utilized for epidemiological studies upon formal request to the NHIS.

### 2.2 Study Population

We identified 2 616 828 individuals with T2DM who underwent a national health examination between 1 January 2015 and 31 December 2016. T2DM was defined based on fasting glucose ≥ 126 mg/dL or ICD‐10‐CM codes E11–14 with antidiabetic prescriptions. After excluding those < 20 years (*n* = 323), with missing variables (*n* = 88 559), or who died within 1 year to minimize reverse causality (*n* = 19 970), 2 507 976 participants were included in the final analysis (Figure S1).

This study adhered to the tenets of the Declaration of Helsinki and was approved by the Kangbuk Samsung Medical Center Institutional Review Board (KBSMC 2025‐02‐019), Seoul, Korea. The Institutional Review Board waived the requirement for informed consent as de‐identified information was used for the analyses.

### 2.3 Definition of BMI Status and Underweight

During the health examination, height and weight were measured, and BMI was calculated by dividing weight (kg) by height (m) squared. The participants were then categorized according to their BMI using the WHO Western Pacific Region guideline strata of underweight (< 18.5 kg/m^2^), normal weight (18.5–22.9 kg/m^2^) and overweight and obesity (≥ 23.0 kg/m^2^) accounting for their Asian ethnicity [16]. The underweight group was further categorized into three subgroups: mild underweight (17.0 ≤ BMI < 18.5 kg/m^2^), moderate underweight (16.0 ≤ BMI < 17.0 kg/m^2^) and severe underweight (BMI < 16.0 kg/m^2^) [17]. The categorization of overweight and obesity followed the same guideline: overweight (23.0 ≤ BMI < 25.0 kg/m^2^), Class I obesity (25.0 ≤ BMI < 30.0 kg/m^2^), Class II obesity (30.0 ≤ BMI < 35.0 kg/m^2^) and Class III obesity (BMI ≥ 35.0 kg/m^2^).

### 2.4 Mortality Outcome

The primary study outcome was mortality from all gastrointestinal cancers, including oesophageal, gastric, small intestine, colorectal and anal cancer. Additionally, mortality for each specific cancer type within this category was individually assessed. Mortality from cancer was evaluated using national death records, with all outcomes identified based on ICD‐10‐CM codes. The specific cancers and their corresponding ICD‐10‐CM codes are as follows: all gastrointestinal cancers (C15–21), oesophageal cancer (C15), gastric cancer (C16), small intestine cancer (C17), colorectal cancer (C18–20) and anal cancer (C21). Follow‐up was conducted until 31 December 2022, to ensure comprehensive assessment of long‐term mortality outcomes.

### 2.5 Definitions of Covariates and Comorbidities

All covariates included in the analysis were obtained at the time of study enrollment. Participants with an income in the lowest 25th percentile of the population were classified as having low income. Smoking status was categorized into three groups: never smokers, ex‐smokers and current smokers. Alcohol consumption was classified based on daily intake, with mild drinking defined as consuming less than 30 g per day for males or less than 20 g per day for females, while heavy drinking was defined as consuming 30 g or more per day for males or 20 g or more per day for females. Physical activity levels were assessed through self‐reported questionnaires administered during health examinations. Participants were considered physically active if they engaged in high‐intensity exercise for at least 20 min on three or more days per week or participated in moderate‐intensity exercise for at least 30 min on five or more days per week [18, 19].

Medical comorbidities were defined as follows. Hypertension was diagnosed based on either a systolic blood pressure of 140 mmHg or higher, a diastolic blood pressure of 90 mmHg or higher, or a history of antihypertensive medication claims recorded under ICD‐10‐CM codes I10–13 and I15. Dyslipidemia was identified in participants with a total cholesterol level of 240 mg/dL or higher or those with a documented history of dyslipidemia medication claims under ICD‐10‐CM code E78. Chronic kidney disease (CKD) was defined as an estimated glomerular filtration rate of less than 60 mL/min/1.73 m^2^, calculated using the Modification of Diet in Renal Disease equation. A history of cancers other than gastrointestinal cancer was determined based on NHIS records and ICD‐10‐CM codes, with only events occurring before the screening date being considered. Information on diabetes duration, insulin medication and the number of oral antidiabetic drugs (OADs) prescribed was collected from medication claim records (ICD‐10‐CM codes E11–14) and antidiabetic prescription records.

### 2.6 Statistical Analysis

Baseline characteristics were summarized as frequencies with percentages for categorical variables and means with standard deviations for continuous variables. Between‐group differences were assessed using chi‐square tests and one‐way analysis of variance. Gastrointestinal cancer mortality risk was evaluated using the Cox proportional hazards models, reported as hazard ratios (HRs) with 95% confidence intervals (CIs); these models estimate cause‐specific HRs, in which deaths from causes other than gastrointestinal cancer were treated as censored observations. Model 1 was unadjusted, Model 2 was adjusted for age and sex and Model 3 was additionally adjusted for smoking, alcohol consumption, physical activity, income, hypertension, dyslipidemia, CKD, fasting glucose, duration of T2DM, insulin use and the use of multiple oral antidiabetic drugs. The proportional hazards assumption for BMI status was evaluated using log‐minus‐log plots derived from unadjusted Kaplan–Meier curves, rather than formal statistical tests (e.g., Schoenfeld residuals) or plots based on adjusted Cox models, as such tests are prone to detecting statistically significant but clinically negligible deviations in large sample sizes such as ours, and adjusted plots would themselves presuppose the proportional hazards assumption being tested.

To assess nonlinear relationships, BMI was also analysed as a continuous variable using penalized spline regression. Kaplan–Meier curves were generated to compare cumulative incidence across BMI categories. For categorical comparisons, the BMI 25.0–29.9 kg/m^2^ group was selected as the reference, based on prior general population studies identifying this range as the nadir of cancer mortality risk [4]. In analyses of underweight, the nonunderweight group (BMI ≥ 18.5 kg/m^2^) was used as the reference.

Subgroup analyses were conducted by age, sex, smoking status, CKD and history of nongastrointestinal cancer. Sensitivity analyses included competing risk models using Fine‐Gray subdistribution hazard models, in which all deaths other than gastrointestinal cancer death were treated as competing events and retained in the risk set, in contrast to the standard Cox proportional hazards models used in the primary analysis, which treated these deaths as censored observations. Additional sensitivity analyses applying 2‐, 3‐ and 4‐year lag periods were performed, excluding early deaths to further minimize the potential impact of reverse causality. To clarify whether the elevated gastrointestinal cancer mortality risk observed in underweight individuals reflects a higher risk of developing gastrointestinal cancer or an adverse effect of underweight status after cancer onset, gastrointestinal cancer incidence was additionally examined across BMI categories, excluding small intestine and anal cancer owing to the small number of incident cases. Statistical analyses were performed using SAS version 9.4 (SAS Institute Inc., Cary, NC). All *p*‐values were two‐sided, and statistical significance was set at *p* < 0.05.

## 3 Results

### 3.1 Baseline Characteristics of Study Population by BMI Status

A total of 2 507 976 participants were included in the final analysis. Baseline characteristics stratified by BMI groups are shown in Table 1. Among all participants, 34 159 individuals (1.4%) were classified as underweight. Regarding age distribution, 65.5% of participants were younger than 65 years. The severe underweight group had the highest proportions of low‐income individuals and nondrinkers, whereas the overweight group showed the highest prevalence of regular exercise. Hypertension and dyslipidemia were the most prevalent in Classes III and II obesity, respectively, while CKD was the most frequent in severe underweight. Lastly, 4.2% of all participants had a history of cancer, excluding gastrointestinal cancer. Additional baseline characteristics focusing on underweight and nonunderweight are presented in Table S1.

**TABLE 1:** Baseline characteristics of the study participants stratified by BMI status.

	Total	BMI group								*p*
		Underweight			Normal weight	Overweight	Obesity			
		Severe	Moderate	Mild			Class I	Class II	Class III	
		< 16.0 kg/m^2^	16.0–16.9 kg/m^2^	17.0–18.4 kg/m^2^	18.5–22.9 kg/m^2^	23.0–24.9 kg/m^2^	25.0–29.9 kg/m^2^	30.0–34.9 kg/m^2^	≥ 35.0 kg/m^2^	
Number	2 507 976	2568	5444	26 147	586 301	606 198	1 045 331	202 862	33 125	
Age (years)	59.6 ± 12.0	65.4 ± 15.3	63.4 ± 15.0	62.1 ± 14.2	61.61 ± 11.84	60.86 ± 11.25	58.99 ± 11.65	54.48 ± 12.81	48.82 ± 13.46	< 0.0001
< 65	1 643 495 (65.5)	1198 (46.7)	2830 (52.0)	14 620 (55.9)	350 716 (59.8)	380 169 (62.7)	708 791 (67.8)	156 693 (77.2)	28 478 (86.0)	
≥ 65	864 481 (34.5)	1370 (53.3)	2614 (48.0)	11 527 (44.1)	235 585 (40.2)	226 029 (37.3)	336 540 (32.2)	46 169 (22.8)	4647 (14.0)	
Sex (male)	1 510 574 (60.2)	1272 (49.5)	2854 (52.4)	13 986 (53.5)	333 969 (57.0)	379 197 (62.6)	653 829 (62.6)	110 219 (54.3)	15 248 (46.0)	< 0.0001
Height (cm)	162.8 ± 9.4	159.5 ± 9.7	160.3 ± 9.4	160.9 ± 9.2	161.84 ± 9	162.82 ± 9.1	163.26 ± 9.42	163.28 ± 10.18	163.28 ± 11.05	< 0.0001
Weight (kg)	67.4 ± 12.6	38.6 ± 5.1	42.6 ± 5.1	46.4 ± 5.4	56.3 ± 7.04	63.77 ± 7.28	72.06 ± 9.04	84.73 ± 11.14	100.56 ± 15.02	< 0.0001
BMI (kg/m^2^)	25.3 ± 3.5	15.1 ± 0.8	16.5 ± 0.3	17.9 ± 0.4	21.42 ± 1.13	23.97 ± 0.57	26.95 ± 1.35	31.66 ± 1.32	37.56 ± 2.67	< 0.0001
Social history										
Low‐income level (*n*, %)	540 433 (21.6)	731 (28.5)	1486 (27.3)	6759 (25.9)	131 610 (22.5)	127 358 (21.0)	219 008 (21.0)	45 274 (22.3)	8207 (24.8)	< 0.0001
Regular exercise (*n*, %)	543 869 (21.7)	264 (10.3)	817 (15.0)	4629 (17.7)	133 526 (22.8)	141 622 (23.4)	222 814 (21.3)	35 302 (17.4)	4895 (14.8)	< 0.0001
Smoking (*n*, %)										< 0.0001
Never smoker	1 380 031 (55.0)	1560 (60.8)	3178 (58.4)	15 054 (57.6)	335 147 (57.2)	328 149 (54.1)	560 061 (53.6)	116 323 (57.3)	20 559 (62.1)	
Ex‐smoker	563 231 (22.5)	299 (11.6)	710 (13.0)	3688 (14.1)	113 115 (19.3)	143 724 (23.7)	256 941 (24.6)	39 999 (19.7)	4755 (14.4)	
Current smoker	564 714 (22.5)	709 (27.6)	1556 (28.6)	7405 (28.3)	138 039 (23.5)	134 325 (22.2)	228 329 (21.8)	46 540 (22.9)	7811 (23.6)	
Drinking (*n*, %)										< 0.0001
None	1 450 549 (57.8)	1884 (73.4)	3821 (70.2)	17 401 (66.6)	362 380 (61.8)	349 200 (57.6)	579 349 (55.4)	116 340 (57.4)	20 174 (60.9)	
Mild	829 816 (33.1)	507 (19.7)	1252 (23.0)	6815 (26.1)	178 839 (30.5)	204 412 (33.7)	361 559 (34.6)	66 250 (32.7)	10 182 (30.7)	
Heavy	227 611 (9.1)	177 (6.9)	371 (6.8)	1931 (7.39)	45 082 (7.7)	52 586 (8.7)	104 423 (10.0)	20 272 (10.0)	2769 (8.4)	
Medical history										
Hypertension (*n*, %)	1 558 506 (62.1)	1134 (44.2)	2413 (44.3)	11 354 (43.4)	306 638 (52.3)	362 594 (59.8)	700 075 (67.0)	148 912 (73.4)	25 386 (76.6)	< 0.0001
Dyslipidemia (*n*, %)	1 458 463 (58.2)	848 (33.0)	1841 (33.8)	9863 (37.7)	307 374 (52.4)	352 038 (58.1)	638 973 (61.1)	127 796 (63.0)	19 730 (59.6)	< 0.0001
Chronic kidney disease (*n*, %)	273 902 (10.9)	417 (16.2)	794 (14.6)	3331 (12.7)	66 779 (11.4)	67 563 (11.2)	113 322 (10.8)	19 230 (9.5)	2466 (7.4)	< 0.0001
Cancer other than GI cancer (*n*, %)	104 918 (4.2)	138 (5.4)	374 (6.9)	1572 (6.0)	28 266 (4.8)	25 819 (4.3)	40 351 (3.9)	7291 (3.6)	1107 (3.3)	< 0.0001
Diabetes duration (*n*, %)										< 0.0001
New onset	757 439 (30.2)	871 (33.9)	1765 (32.4)	8113 (31.0)	154 472 (26.4)	169 605 (28.0)	333 665 (31.9)	74 929 (36.9)	14 019 (42.3)	
< 5 years	594 245 (23.7)	488 (19.0)	983 (18.1)	4689 (17.9)	119 388 (20.4)	138 282 (22.8)	263 351 (25.2)	57 211 (28.2)	9853 (29.7)	
< 10 years	523 667 (20.9)	497 (19.4)	1013 (18.6)	4932 (18.9)	122 550 (20.9)	130 626 (21.6)	219 477 (21.0)	39 124 (19.3)	5448 (16.5)	
≥ 10 years	632 625 (25.2)	712 (27.7)	1683 (30.9)	8413 (32.2)	189 891 (32.4)	167 685 (27.7)	228.838 (21.9)	31 598 (15.6)	3805 (11.5)	
≥ 3 oral antidiabetic drugs (*n*, %)	595 922 (23.8)	475 (18.5)	1162 (21.3)	5847 (22.4)	141 022 (24.1)	143 795 (23.7)	246 332 (23.6)	49 246 (24.3)	8043 (24.3)	< 0.0001
Insulin medication (*n*, %)	213 924 (8.5)	378 (14.7)	827 (15.2)	3727 (14.3)	60 021 (10.24)	50 883 (8.39)	80 561 (7.71)	15 128 (7.46)	2399 (7.24)	< 0.0001
Metabolic profile										
SBP (mmHg)	128.5 ± 15.1	119.4 ± 17.1	120.5 ± 16.9	121.9 ± 16.5	125.4 ± 15.4	127.8 ± 14.7	129.9 ± 14.6	132.5 ± 14.9	135.7 ± 16.1	< 0.0001
DBP (mmHg)	78.1 ± 10.0	73.64 ± 10.7	73.9 ± 10.6	74.2 ± 10.3	75.7 ± 9.8	77.3 ± 9.6	79.1 ± 9.7	81.5 ± 10.3	84.1 ± 11.4	< 0.0001
Fasting glucose (mg/dL)	144.5 ± 45.8	153.9 ± 75.3	150.7 ± 68.4	149.0 ± 62.7	144.6 ± 50.0	143.5 ± 44.8	144.2 ± 43.1	147.3 ± 44.7	151.1 ± 48.2	< 0.0001
Total cholesterol (mg/dL)	185.2 ± 43.7	176.2 ± 42.3	176.6 ± 41.6	177.2 ± 41.0	181.7 ± 42.7	184.1 ± 43.5	186.8 ± 44.1	190.5 ± 44.5	193.9 ± 44.0	< 0.0001
LDL‐C (mg/dL)	103.1 ± 38.4	94.5 ± 36.3	94.8 ± 35.6	96.1 ± 35.9	101.2 ± 37.5	102.8 ± 38.3	103.8 ± 38.8	105.9 ± 39.4	109.6 ± 39.3	< 0.0001
HDL‐C (mg/dL)	51.0 ± 14.7	59.7 ± 19.1	59.9 ± 21.5	58.9 ± 17.5	53.8 ± 16.0	51.0 ± 14.7	49.5 ± 13.7	48.9 ± 13.5	48.8 ± 14.0	< 0.0001
Triglyceride^a^ (mg/dL)	137.7 (137.6, 137.8)	96.5 (94.6, 98.3)	95.1 (93.7, 96.4)	95.5 (94.9, 96.1)	116.0 (115.9, 116.2)	134.1 (133.9, 134.3)	149.9 (149.7, 150.0)	163.0 (162.6, 163.3)	164.1 (163.2, 165.0)	< 0.0001
eGFR (mL/min/1.73m^2^)	84.1 ± 20.2	82.6 ± 22.7	83.8 ± 22.7	84.6 ± 21.9	83.8 ± 20.2	83.3 ± 19.8	83.9 ± 20.0	87.1 ± 21.2	92.4 ± 21.7	< 0.0001
Haemoglobin (g/dL)	14.1 ± 1.7	12.7 ± 1.9	12.9 ± 1.9	13.2 ± 1.8	13.7 ± 1.7	14.1 ± 1.7	14.3 ± 1.7	14.4 ± 1.7	14.4 ± 1.8	< 0.0001
AST (U/L)	29.9 ± 22.2	33.3 ± 39.0	32.6 ± 43.4	30.6 ± 37.9	27.4 ± 23.9	28.0 ± 19.6	30.8 ± 20.6	36.1 ± 25.0	41.6 ± 29.4	< 0.0001
ALT (U/L)	31.9 ± 26.8	23.3 ± 29.3	23.5 ± 27.5	23.1 ± 25.9	25.0 ± 22.3	28.7 ± 22.8	34.6 ± 26.8	44.6 ± 35.7	54.3 ± 44.1	< 0.0001
r‐GTP (U/L)	53.1 ± 75.5	66.2 ± 142.3	62.0 ± 135.4	56.6 ± 126.3	47.7 ± 89.3	49.3 ± 72.0	55.9 ± 68.2	63.0 ± 66.4	65.1 ± 61.0	< 0.0001

*Note:* Data are presented as mean ± standard deviation.

Abbreviations: ALT, alanine aminotransferase; AST, aspartate aminotransferase; BMI, body mass index; DBP, diastolic blood pressure; GI, gastrointestinal; HDL‐C, high‐density lipoprotein cholesterol; LDL‐C, low‐density lipoprotein cholesterol; r‐GTP, gamma‐glutamyl transferase; SBP, systolic blood pressure.

^a^ Geometric mean (95% confidence interval) or number (%).

### 3.2 Mortality Risk From Gastrointestinal Cancers Across the Full Range of BMI Status

During a mean follow‐up period of 5.75 years, 10 978 participants died from gastrointestinal cancer (Table 2). Of these deaths, 975 deaths were attributed to oesophageal cancer, 4391 to gastric cancer, 5317 to colorectal cancer and 295 to other gastrointestinal cancers (255 to small intestine cancer and 40 to anal cancer; Table S2).

**TABLE 2:** Risk of mortality from gastrointestinal cancer across the full range of BMI status in individuals with T2DM.

						Hazard ratios (95% CI)		
	BMI (kg/m^2^)	*N*	Events	Person‐years	Incidence rate^a^	Model 1^b^	Model 2^c^	Model 3^d^
All gastrointestinal cancers	< 16	2568	37	11637.92	3.18	5.39 (3.90–7.44)	3.44 (2.49–4.76)	2.96 (2.14–4.09)
	16–16.9	5444	66	26901.92	2.45	4.13 (3.24–5.27)	2.86 (2.24–3.64)	2.51 (1.96–3.20)
	17–18.4	26 147	309	137515.94	2.25	3.77 (3.36–4.23)	2.81 (2.50–3.15)	2.54 (2.26–2.85)
	18.5–22.9	586 301	3603	3321489.92	1.08	1.81 (1.73–1.90)	1.49 (1.42–1.56)	1.45 (1.38–1.52)
	23–24.9	606 198	2696	3498210.33	0.77	1.29 (1.22–1.35)	1.11 (1.06–1.17)	1.11 (1.06–1.17)
	25–29.9	1 045 331	3635	6063099.56	0.60	1 (ref.)	1 (ref.)	1 (ref.)
	30–34.9	202 862	568	1176122.88	0.48	0.81 (0.74–0.88)	1.20 (1.10–1.31)	1.18 (1.08–1.29)
	≥ 35	33 125	64	191022.86	0.34	0.56 (0.44–0.72)	1.402 (1.10–1.80)	1.35 (1.05–1.73)
Oesophageal cancer	< 16	2568	9	11637.92	0.77	18.12 (9.33–35.22)	15.08 (7.75–29.36)	13.23 (6.77–25.85)
	16–16.9	5444	12	26901.92	0.45	10.40 (5.83–18.54)	8.57 (4.80–15.30)	7.66 (4.28–13.74)
	17–18.4	26 147	38	137515.94	0.28	6.41 (4.56–9.01)	5.32 (3.78–7.49)	4.91 (3.47–6.94)
	18.5–22.9	586 301	391	3321489.92	0.12	2.72 (2.32–3.18)	2.32 (1.98–2.72)	2.29 (1.95–2.69)
	23–24.9	606 198	242	3498210.33	0.07	1.60 (1.34–1.90)	1.37 (1.15–1.63)	1.38 (1.16–1.65)
	25–29.9	1 045 331	263	6063099.56	0.04	1 (ref.)	1 (ref.)	1 (ref.)
	30–34.9	202 862	20	1176122.88	0.02	0.39 (0.25–0.62)	0.65 (0.41–1.02)	0.64 (0.40–1.01)
	≥ 35	33 125	0	191022.86	0.00	N/A	N/A	N/A
Gastric cancer	< 16	2568	13	11637.92	1.12	4.73 (2.74–8.17)	3.09 (1.79–5.33)	2.60 (1.50–4.49)
	16–16.9	5444	26	26901.92	0.97	4.09 (2.77–6.03)	2.86 (1.94–4.22)	2.45 (1.66–3.62)
	17–18.4	26 147	147	137515.94	1.07	4.51 (3.81–5.35)	3.39 (2.86–4.02)	2.99 (2.52–3.55)
	18.5–22.9	586 301	1505	3321489.92	0.45	1.91 (1.78–2.05)	1.60 (1.46–1.69)	1.51 (1.40–1.63)
	23–24.9	606 198	1028	3498210.33	0.29	1.24 (1.14–1.34)	1.07 (0.98–1.16)	1.06 (0.98–1.15)
	25–29.9	1 045 331	1439	6063099.56	0.24	1 (ref.)	1 (ref.)	1 (ref.)
	30–34.9	202 862	211	1176122.88	0.18	0.76 (0.66–0.87)	1.14 (0.99–1.32)	1.13 (0.98–1.31)
	≥ 35	33 125	22	191022.86	0.12	0.49 (0.32–0.74)	1.26 (0.83–1.92)	1.23 (0.81–1.87)
Colorectal cancer	< 16	2568	14	11637.92	1.20	4.09 (2.42–6.91)	2.45 (1.45–4.14)	2.15 (1.27–3.63)
	16–16.9	5444	26	26901.92	0.97	3.25 (2.21–4.79)	2.15 (1.46–3.16)	1.92 (1.30–2.83)
	17–18.4	26 147	118	137515.94	0.86	2.87 (2.38–3.45)	2.08 (1.72–2.50)	1.91 (1.58–2.31)
	18.5–22.9	586 301	1621	3321489.92	0.49	1.62 (1.52–1.73)	1.32 (1.23–1.41)	1.30 (1.21–1.39)
	23–24.9	606 198	1346	3498210.33	0.38	1.28 (1.19–1.37)	1.11 (1.03–1.19)	1.11 (1.04–1.20)
	25–29.9	1 045 331	1829	6063099.56	0.30	1 (ref.)	1 (ref.)	1 (ref.)
	30–34.9	202 862	324	1176122.88	0.28	0.91 (0.81–1.03)	1.32 (1.17–1.49)	1.29 (1.14–1.45)
	≥ 35	33 125	39	191022.86	0.20	0.68 (0.49–0.93)	1.60 (1.17–2.20)	1.52 (1.10–2.08)

Abbreviations: BMI, body mass index; 95% CI, 95% confidence interval; T2DM, type 2 diabetes mellitus.

^a^ Incidence rate per 1000 person‐years.

^b^ Model 1: Nonadjusted.

^c^ Model 2: Adjusted for age, sex.

^d^ Model 3: Adjusted for factors in Model 2 and income, smoking, drinking, physical activity, hypertension, dyslipidemia, chronic kidney disease, fasting glucose, ≥ 3 oral antidiabetic drugs, insulin medication, and T2DM duration.

In the fully adjusted model (Model 3) for all gastrointestinal cancers, the reference group exhibited the lowest adjusted hazard ratio (aHR), while both underweight and obesity were associated with increased mortality risk (Table 2). The highest aHR was observed in the severe underweight group (BMI < 16.0 kg/m^2^), reaching 2.96 (95% CI 2.14–4.09), nearly threefold higher than the reference group, and exceeding the aHR of the Class III obesity group (1.35, 95% CI 1.05–1.73). Both underweight and obesity showed a severity‐related pattern, yielding a characteristic reverse J‐shaped association. This pattern was more clearly visualized in the penalized spline curve shown in Figure 1A.

**FIGURE 1:**
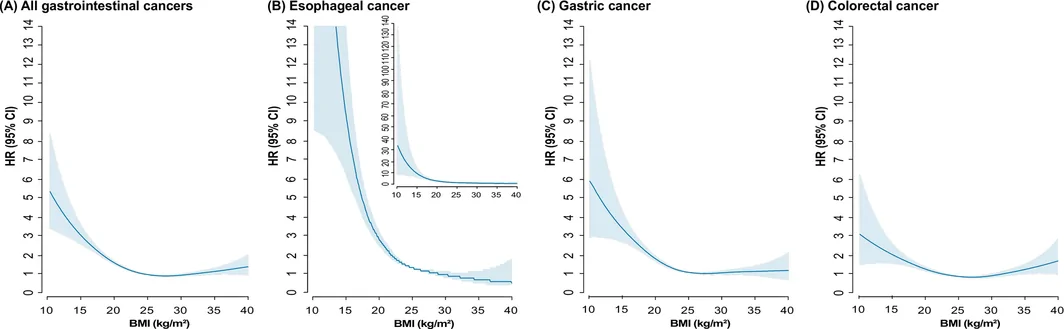
Penalized spline analysis of association between BMI and mortality from (A) all gastrointestinal cancers, (B) oesophageal cancer, (C) gastric cancer and (D) colorectal cancer among individuals with T2DM. Age, sex, income, smoking, drinking, physical activity, hypertension, dyslipidemia, chronic kidney disease, fasting glucose, ≥ 3 oral antidiabetic drugs, insulin use and diabetes duration were adjusted. BMI, body mass index; 95% CI, 95% confidence interval; HR, hazard ratio; T2DM, type 2 diabetes mellitus.

When stratified by major gastrointestinal cancers—oesophageal, gastric and colorectal cancer—overall trends were similar but exhibited subtle variations (Table 2). For oesophageal cancer, severe underweight group showed the highest mortality risk, with an aHR of 13.23 (95% CI 6.77–25.85), with risk rising consistently with greater underweight severity. However, the Class II obesity group showed a trend toward lower mortality (aHR 0.64, 95% CI 0.40–1.01) without statistical significance, and no mortality events were recorded in the Class III obesity group. The spline curve demonstrated a sharp increase in risk with increasing underweight severity, while risk appeared to decrease modestly across the obesity range (Figure 1B). For gastric cancer, the overall trend was similar; however, the mild underweight group demonstrated the highest aHR at 2.99 (95% CI 2.52–3.55), followed by the severe underweight group at 2.60 (95% CI 1.50–4.49), with no significant association observed in obesity groups (Figure 1C). For colorectal cancer, the highest risk was observed in the severe underweight group, with an aHR of 2.15 (95% CI 1.27–3.63), representing the lowest peak risk among the three major cancers. Notably, unlike other cancers, both Classes II and III obesity groups exhibited significantly elevated aHRs, with the Class III obesity group reaching 1.52 (95% CI 1.10–2.08). These differences were evident in the spline curve (Figure 1D).

For small intestine and anal cancers, the number of mortality events was very low, and some BMI groups for anal cancer had no recorded events (Table S2). Accordingly, no significant differences in mortality risk across BMI groups were observed for small intestine or anal cancer, except for a significantly elevated aHR of 5.20 (95% CI 1.14–23.75) observed in the mild underweight group for anal cancer.

### 3.3 Incidence of Gastrointestinal Cancers According to BMI Status

During follow‐up, crude incidence rates of all gastrointestinal cancers were numerically higher in the underweight groups than in the reference group (severe underweight, 4.60; moderate underweight, 4.25; mild underweight, 4.39; reference group, 3.76 per 1000 person‐years; Table S3). However, after full adjustment, no significant association was observed between underweight and the incidence of all gastrointestinal cancers (severe underweight aHR 0.98, 95% CI 0.74–1.31; moderate underweight aHR 0.96, 95% CI 0.79–1.17; mild underweight aHR 1.03, 95% CI 0.95–1.12) nor for gastric or colorectal cancer incidence individually. In contrast, mild underweight remained significantly associated with an increased incidence of oesophageal cancer (aHR 3.38, 95% CI 2.60–4.39), whereas Classes II and III obesity were significantly associated with an increased incidence of colorectal cancer (aHR 1.10 [95% CI 1.05–1.15] and 1.17 [95% CI 1.04–1.32], respectively).

### 3.4 Mortality Risk From Gastrointestinal Cancers in Underweight Versus Nonunderweight Individuals

To further investigate the elevated mortality risk in underweight groups compared to the nonunderweight group, additional analyses were conducted for all gastrointestinal cancers, oesophageal cancer, gastric cancer and colorectal cancer (Table 3 and Figure 2).

**TABLE 3:** Risk of mortality from gastrointestinal cancer according to underweight status in individuals with T2DM.

						Hazard ratios (95% CI)		
	BMI (kg/m^2^)	*N*	Events	Person‐years	Incidence rate^a^	Model 1^b^	Model 2^c^	Model 3^d^
All gastrointestinal cancers	Severe underweight (< 16.0)	2568	37	11 638	3.18	4.35 (3.15–6.01)	2.90 (2.10–4.01)	2.46 (1.78–3.41)
	Moderate underweight (16.0–16.9)	5444	66	26 902	2.45	3.34 (2.62–4.25)	2.42 (1.90–3.08)	2.09 (1.64–2.67)
	Mild underweight (17–18.4)	26 147	309	137 516	2.25	3.05 (2.72–3.41)	2.38 (2.12–2.67)	2.13 (1.90–2.38)
	Nonunderweight (18.5 ≤)	2 473 817	10 566	14 249 946	0.74	1 (ref.)	1 (ref.)	1 (ref.)
Oesophageal cancer	Severe underweight (< 16.0)	2568	9	11 638	0.77	12.22 (6.34–23.57)	10.25 (5.31–19.79)	8.47 (4.38–16.40)
	Moderate underweight (16.0–16.9)	5444	12	26 902	0.45	7.01 (3.97–12.39)	5.84 (3.30–10.34)	4.95 (2.79–8.77)
	Mild underweight (17–18.4)	26 147	38	137 516	0.28	4.32 (3.13–5.98)	3.65 (2.64–5.05)	3.19 (2.30–4.43)
	Nonunderweight (18.5 ≤)	2 473 817	916	14 249 946	0.06	1 (ref.)	1 (ref.)	1 (ref.)
Gastric cancer	Severe underweight (< 16.0)	2568	13	11 638	1.12	3.81 (2.21–6.56)	2.58 (1.50–4.45)	2.15 (1.25–3.71)
	Moderate underweight (16.0–16.9)	5444	26	26 902	0.97	3.29 (2.24–4.83)	2.40 (1.63–3.53)	2.04 (1.39–3.00)
	Mild underweight (17–18.4)	26 147	147	137 516	1.07	3.63 (3.08–4.28)	2.85 (2.42–3.36)	2.49 (2.11–2.94)
	Nonunderweight (18.5 ≤)	2 473 817	4205	14 249 946	0.30	1 (ref.)	1 (ref.)	1 (ref.)
Colorectal cancer	Severe underweight (< 16.0)	2568	14	11 638	1.20	3.40 (2.02–5.75)	2.13 (1.26–3.60)	1.87 (1.11–3.16)
	Moderate underweight (16.0–16.9)	5444	26	26 902	0.97	2.71 (1.84–3.98)	1.89 (1.28–2.77)	1.68 (1.14–2.46)
	Mild underweight (17–18.4)	26 147	118	137 516	0.86	2.39 (1.99–2.87)	1.83 (1.52–2.19)	1.67 (1.39–2.01)
	Nonunderweight (18.5 ≤)	2 473 817	5159	14 249 946	0.36	1 (ref.)	1 (ref.)	1 (ref.)

Abbreviations: BMI, body mass index; 95% CI, 95% confidence interval; T2DM, type 2 diabetes mellitus.

^a^ Incidence rate per 1000 person‐years.

^b^ Model 1: Nonadjusted.

^c^ Model 2: Adjusted for age, sex.

^d^ Model 3: Adjusted for factors in Model 2 and income, smoking, drinking, physical activity, Hypertension, dyslipidemia, chronic kidney disease, fasting glucose, ≥ 3 oral antidiabetic drugs, insulin medication, and T2DM duration.

**FIGURE 2:**
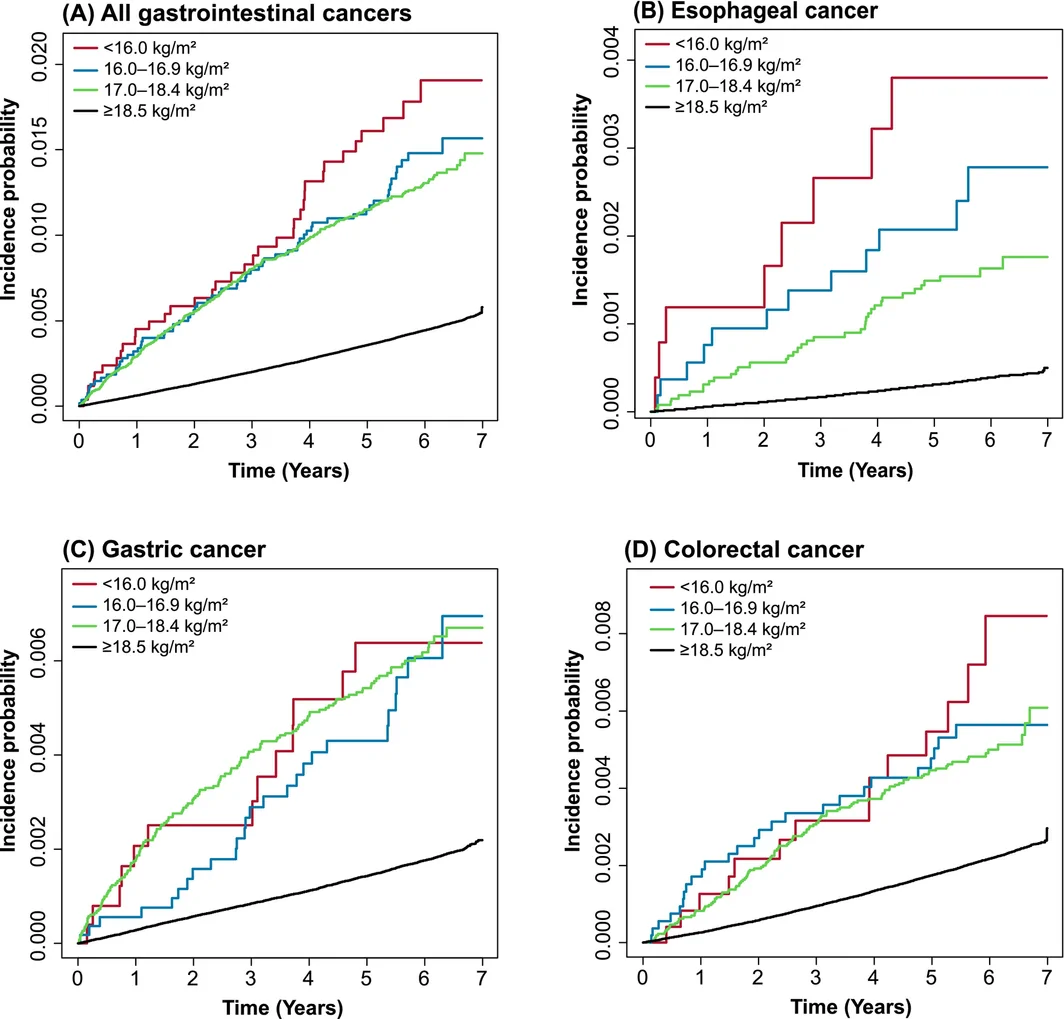
Kaplan–Meier survival curves for (A) all gastrointestinal cancers, (B) oesophageal cancer, (C) gastric cancer and (D) colorectal cancer by degree of underweight. All log‐rank *p*‐value < 0.001.

When compared with the nonunderweight group (BMI ≥ 18.5 kg/m^2^), significantly increased aHRs were observed across all underweight groups in all models. The patterns of association according to the severity of underweight were consistent with the results presented in Table 2. The severe underweight group exhibited the highest aHR for all gastrointestinal cancers (2.46; 95% CI, 1.78–3.41), oesophageal cancer (8.47; 95% CI, 4.38–16.40) and colorectal cancer (1.87; 95% CI, 1.11–3.16). For gastric cancer, however, the mild underweight group demonstrated the highest risk, with an aHR of 2.49 (95% CI, 2.11–2.94).

The Kaplan–Meier curves illustrating cumulative incidence of mortality events for each cancer outcome also indicated significantly increased cumulative incidence probabilities among underweight groups (Figure 2).

### 3.5 Subgroup Analyses for Mortality From Gastrointestinal Cancers

Further subgroup analyses were conducted to assess the impact of underweight status on gastrointestinal cancer mortality across various clinical and demographic subgroups (Figure 3 and Table S4–S7). Although the degree of impact varied across subgroups, the overall trend remained consistent.

**FIGURE 3:**
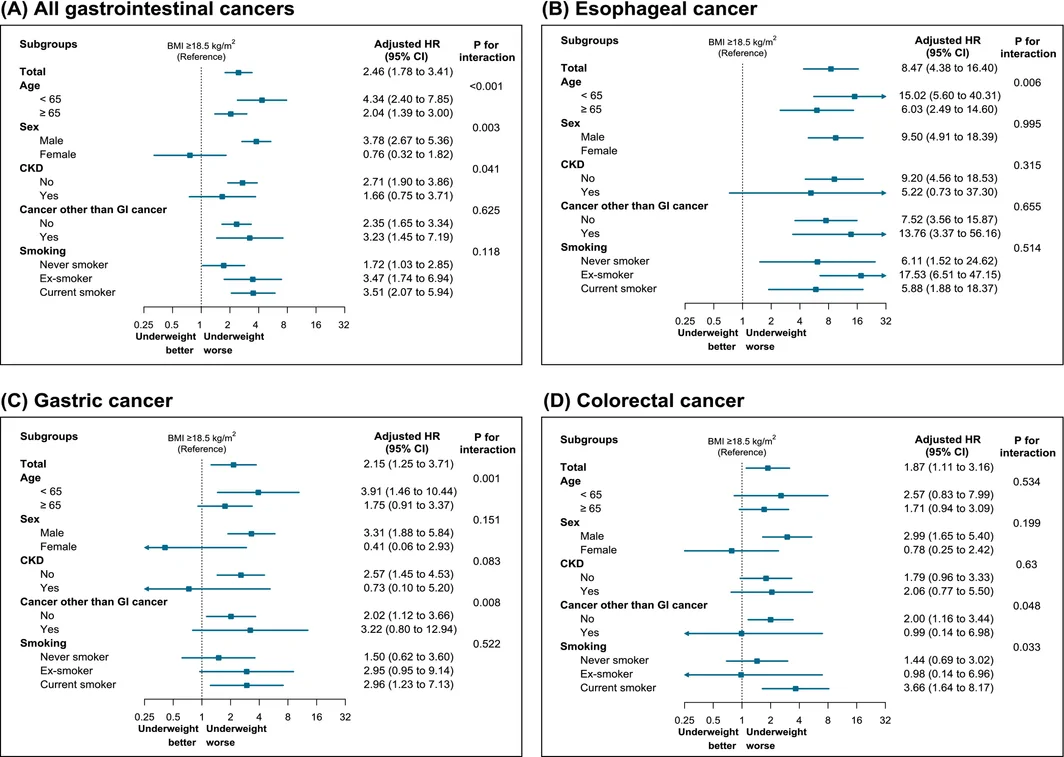
Stratified analyses for (A) all gastrointestinal cancers, (B) oesophageal cancer, (C) gastric cancer and (D) colorectal cancer mortality risk according to age, sex, presence of CKD, history of cancer other than gastrointestinal cancer and smoking status. Hazard ratios (95% CIs) adjusted for age, sex, income, smoking, drinking, physical activity, hypertension, dyslipidemia, chronic kidney disease, fasting glucose, ≥ 3 oral antidiabetic drugs, insulin use and diabetes duration are presented. BMI, body mass index; 95% CI, 95% confidence interval; CKD, chronic kidney disease.

For gastrointestinal cancers, the incidence rate was lower in individuals under 65 years of age compared to those aged 65 years or older. However, the increase in mortality risk associated with underweight status was significantly higher in this younger subgroup, with the severe underweight group exhibiting the most pronounced increase in risk (aHR 4.34; 95% CI, 2.40–7.85). A similar significant difference by age was observed for oesophageal and gastric cancer but not for colorectal cancer.

Additionally, for all gastrointestinal cancers mortality, males showed both higher incidence rates and more pronounced mortality risk with severe underweight (aHR, 3.78; 95% CI, 2.67–5.36), though no significant sex‐based differences emerged when individual cancer types were analysed. A similar pattern was identified in subgroup analyses based on the presence of CKD, with severe underweight significantly increasing mortality risk from all gastrointestinal cancers (aHR, 2.71; 95% CI, 1.90–3.86) in individuals without CKD. In contrast, no significant difference was observed between subgroups stratified by history of nongastrointestinal cancer.

Among smoking strata, all underweight groups showed significantly increased mortality risk compared to the nonunderweight group. However, both current and ex‐smokers tended to have a greater increase in all gastrointestinal cancer mortality risk than never smokers. Specifically, in colorectal cancer, current smokers exhibited a significantly higher relative mortality risk.

### 3.6 Sensitivity Analysis

First, the proportional hazards assumption for BMI status was evaluated using log‐minus‐log plots for all gastrointestinal cancer mortality, which showed reasonably parallel, noncrossing curves across BMI groups (severe underweight, moderate underweight, mild underweight and nonunderweight), particularly during the earlier follow‐up period (Figure S2). Second, a competing risk analysis was performed using Fine‐Gray subdistribution hazard models, in which all deaths other than gastrointestinal cancer death were treated as competing events (Table S8). Compared with the primary Cox models, the magnitude of association was generally attenuated across all cancer types; aHRs for severe underweight were 1.67 (95% CI 1.21–2.32) for all gastrointestinal cancers, 5.82 (95% CI 2.97–11.39) for oesophageal cancer, 1.48 (95% CI 0.85–2.56) for gastric cancer and 1.23 (95% CI 0.73–2.09) for colorectal cancer. Associations remained significant across all underweight subgroups for all gastrointestinal cancers and oesophageal cancer, whereas severe underweight was no longer significantly associated with gastric cancer mortality, and neither severe nor moderate underweight remained significantly associated with colorectal cancer mortality. Third, to further address the possibility of reverse causation, 2‐, 3‐ and 4‐year lag periods were sequentially applied (Tables S9–S11). For all gastrointestinal cancers, oesophageal cancer and gastric cancer, the aHRs for underweight, particularly severe underweight, did not show a consistent pattern of attenuation with increasing lag duration; for example, the aHRs for severe underweight and all gastrointestinal cancer mortality were 2.46, 2.24, 2.64 and 2.92 with 1‐, 2‐, 3‐ and 4‐year lag periods, respectively. For colorectal cancer, the aHRs for underweight generally declined with increasing lag duration. The moderate underweight group was no longer significantly associated from the 2‐year lag period onward (1.68, 1.34, 1.21 and 1.26 with 1‐, 2‐, 3‐ and 4‐year lags), and the mild underweight group declined monotonically and lost significance at the 4‐year lag period (1.67, 1.64, 1.50 and 1.20). In contrast, the estimate for severe underweight remained stable (1.87, 1.84, 1.95, and 2.14).

## 4 Discussion

In this study, we found that mortality risk from all gastrointestinal cancers among individuals with T2DM exhibited its nadir in the Class I obesity group (BMI 25.0–29.9 kg/m^2^), with significantly increased risks observed in both underweight and obesity categories. HRs showed a stronger association between underweight status and mortality, with this association intensifying progressively as underweight severity increased. Interestingly, among major gastrointestinal cancers, the mortality risk from underweight was most pronounced in oesophageal cancer, followed by gastric and colorectal cancers. Conversely, a significant association between obesity and increased mortality risk was observed only in colorectal cancer, whereas in gastric cancer, no association was found, and in oesophageal cancer, obesity showed a trend toward lower mortality without statistical significance. For small intestine and anal cancers, no significant associations were identified, likely due to their low incidence.

In the general population, underweight status has frequently been identified as a significant risk factor for increased cancer mortality, which occasionally exceeds the effects of obesity [1, 2, 4]. A comprehensive meta‐analysis incorporating data from 73 cohort studies reported that underweight was significantly associated with increased mortality from most gastrointestinal cancers, whereas obesity appeared protective, reducing gastrointestinal cancer mortality risk [1]. Specifically, aHR for mortality from all gastrointestinal cancers was 1.46 (95% CI, 1.33–1.60) for underweight and 0.67 (95% CI, 0.56–0.80) for obesity. Another nationwide cohort study has consistently demonstrated a reverse J‐shaped association between BMI and gastrointestinal cancer mortality, which is similar to our findings [4]. Studies of individual gastrointestinal cancers reported mixed findings regarding obesity but consistently confirmed poorer outcomes and increased mortality risks with underweight status [3, 20]. Moreover, underweight status has been linked to not only higher mortality risk but also other adverse outcomes after gastrointestinal cancer surgery [21].

Despite consistent findings in the general population, the rationale for conducting this study stemmed from the well‐documented adverse impact of obesity on clinical outcomes specifically in T2DM, where obesity has been associated with increased risks of cardiovascular mortality [9, 22], cancer [23], infection [24] and all‐cause mortality risk [10, 25]. These associations are believed to arise from pathophysiological mechanisms such as chronic low‐grade inflammation and immunometabolic dysfunction linked to obesity in the context of T2DM [26, 27]. This raised important questions about whether the patterns seen in the general population—particularly the higher cancer mortality risk in underweight relative to obesity—would also hold true in a metabolically distinct T2DM population. Our results were largely consistent with those from the general population, showing a reverse J‐shaped association.

In subgroup analyses, underweight was significantly associated with increased gastrointestinal cancer mortality, particularly in younger individuals under 65 years of age, males and those without CKD. Given that younger individuals and those without CKD generally have a lower baseline risk of cancer mortality, our findings suggest that underweight is associated with increased mortality risk even in the absence of these conventional risk factors [28, 29]. In males, this association is notable considering their higher prevalence of gastrointestinal cancers, highlighting the importance of maintaining an adequate weight [29]. Regarding smoking, although not statistically significant, the impact of underweight on gastrointestinal cancer mortality appeared more pronounced among current and ex‐smokers, especially with severe underweight. However, a significant increase in risk was also observed among never smokers, suggesting that underweight remains an independent risk factor even after accounting for potential confounding by smoking.

An important question is whether the elevated gastrointestinal cancer mortality risk among underweight individuals reflects a higher incidence of gastrointestinal cancer or a poorer prognosis after diagnosis. In an additional incidence analysis, crude incidence rates were numerically higher among underweight individuals, but this difference was largely attenuated after adjustment, with no significant association between underweight and gastrointestinal cancer incidence overall. This contrasts with the twofold to threefold increase in mortality risk observed in the same population, suggesting that the excess mortality more likely reflects factors operating after cancer diagnosis—such as reduced physiological reserve or poorer treatment tolerance—rather than a higher incidence of gastrointestinal cancer itself. Oesophageal cancer was a notable exception, where mild underweight remained significantly associated with increased incidence, suggesting that the underlying mechanism may not be uniform across gastrointestinal cancer subtypes. Notably, even for oesophageal cancer, the magnitude of the mortality association considerably exceeded that of the incidence association, suggesting that postdiagnostic factors still contribute substantially to the excess mortality even where an incidence effect is also present.

Although reverse causation remains an important methodological concern in studies of this kind, several observations from our lag period sensitivity analyses argue against reverse causation as the primary explanation for our findings. If the observed associations were driven mainly by preclinical, undiagnosed cancer at baseline, the strength of association would be expected to attenuate progressively as the lag period lengthened. However, for all gastrointestinal cancers, oesophageal cancer and gastric cancer, this pattern was not observed; associations with underweight, particularly severe underweight, did not decline monotonically and in some instances strengthened with longer lag periods. This pattern suggests that factors other than transient preclinical weight loss, such as sustained nutritional frailty or sarcopenia, may underlie the association for these cancer types. Colorectal cancer followed a different pattern, with the associations for mild and moderate underweight weakening as the lag period was extended, indicating that residual reverse causation cannot be excluded for this cancer type. The estimate for severe underweight nonetheless remained stable across lag periods, which is not the pattern expected if preclinical weight loss were the principal explanation, although the small number of events in this stratum limits interpretation. Attenuation was also observed in the competing risk analysis treating all nongastrointestinal cancer deaths as competing events. This is likely because the subdistribution hazard approach retains individuals who die of competing causes within the risk set, rather than removing them through censoring. As a result, the estimated hazard for gastrointestinal cancer death decreases as the frequency of competing mortality increases. This pattern is particularly relevant to underweight individuals, who carry an elevated risk of death from causes other than gastrointestinal cancer [30]. The cause‐specific and subdistribution hazard estimates should therefore be regarded as complementary rather than contradictory.

The reverse J‐shaped association observed in our study suggests that both underweight and obesity contribute to gastrointestinal cancer mortality through distinct biological mechanisms. In underweight individuals, low BMI may reflect sarcopenia and reduced metabolic reserves, compromising tolerance to cancer treatments such as chemoradiation therapy or surgery [6, 31]. In gastrointestinal cancers, digestive symptoms are frequently observed and often contribute to further weight loss, making preexisting underweight especially detrimental [5]. Since T2DM is a known risk factor for sarcopenia, this association may be strengthened in individuals with both T2DM and underweight [32, 33]. Given these considerations, it is plausible that overweight individuals may have experienced lower mortality risk due to greater metabolic reserves and preserved muscle mass. However, as our study also showed, moderate to severe obesity may increase gastrointestinal cancer mortality risk through adverse mechanisms such as chronic inflammation and adipokine imbalance [34]. Nevertheless, our findings suggest that underweight‐related metabolic frailty and nutritional deficits may exert an even greater influence in T2DM, as similarly reported in the general population.

Interestingly, while a reverse J‐shaped association was observed overall, the magnitude and direction of this association varied across cancer types. For oesophageal cancer, our study identified a different pattern of association compared to other gastrointestinal cancers. Underweight showed the strongest association with increased mortality risk, whereas obesity was not associated with excess risk and instead showed a possible inverse trend. Similar findings have been reported in general population studies, reflecting pathological and etiological features of oesophageal cancer that may explain these differences [4]. Several studies reported that the inverse association was limited to squamous cell carcinoma, with no comparable pattern in adenocarcinoma [35, 36]. This suggests that histological subtype may partially explain the differing associations seen in our analysis. Conversely, colorectal cancer was the only cancer type in which obesity was significantly associated with an increased mortality risk. This may be explained by mechanisms specific to colorectal cancer, where obesity‐related chronic low‐grade inflammation and dysregulated adipokine secretion promote tumour initiation, progression and impaired treatment response [37]. Such mechanisms may underlie the heightened mortality risk in obese individuals with colorectal cancer, distinguishing them from other gastrointestinal cancers. This trend may also explain why the overall reverse J‐shaped association in our study closely mirrored the pattern seen in colorectal cancer, which accounted for most mortality events in the cohort.

The International Agency for Research on Cancer (IARC) has classified numerous gastrointestinal cancers, including oesophageal, gastric and colorectal cancer, as obesity‐related cancers, leading to greater attention on cancer screening for obese populations [38]. Our study found an increased mortality risk in Classes II and III obesity groups, aligning with the IARC findings. However, as the nadir for mortality risk was observed in the reference group with a BMI of 25.0–29.9 kg/m^2^, and risk rose further in underweight group, these findings raise questions about the optimal BMI range for minimizing cancer‐related mortality in T2DM. Furthermore, various cohort studies investigating BMI, cancer prognosis and mortality risk have also identified the nadir at a BMI of 25.0–29.9 kg/m^2^, which is consistent with our results [2, 39, 40]. Based on these findings, it is also critical to maintain weight above the underweight range as a preventive strategy against gastrointestinal cancer mortality. Therefore, existing clinical guidelines recommending only weight loss for individuals with T2DM and cancer may need to be reconsidered [7, 8, 41]. Instead of a generalized emphasis on weight reduction, a more individualized approach incorporating lifestyle modifications and tailored weight management may be more beneficial. Moreover, because the association between BMI and mortality differed across specific gastrointestinal cancers in our study, weight management goals may need to be adapted according to cancer type and individual metabolic characteristics.

This study has several limitations. First, as an observational study, it is inherently limited in its ability to establish a causal relationship between BMI and gastrointestinal cancer mortality. However, we sought to address this limitation by adjusting for a wide range of covariates, including social history and comorbidities, to minimize potential confounding factors. Second, because weight loss can occur as a consequence of gastrointestinal cancer, reverse causation may pose a challenge in interpreting the results. We applied 1‐, 2‐, 3‐ and 4‐year lag periods to address this issue, and the resulting patterns are discussed above; residual reverse causation could not be entirely excluded, particularly for colorectal cancer. Third, when defining gastrointestinal cancer mortality, we relied solely on ICD‐10‐CM codes provided by the NHIS. As a result, the study lacked information on the pathological characteristics and cancer stage at the time of diagnosis, which may have influenced mortality outcomes. Fourth, we were unable to directly examine the association between BMI at the time of cancer diagnosis and subsequent mortality, as health examinations in the NHIS database are not necessarily performed close to the time of diagnosis and BMI is not routinely recorded in cancer treatment‐related claims. Our inference that the excess mortality arises after cancer onset is therefore indirect, and the contributions of stage, treatment intensity and treatment tolerance remain unquantified. Finally, our study relied solely on BMI, which does not distinguish between muscle mass and fat distribution. Given that sarcopenia and body composition are critical determinants of cancer outcomes, the inability to directly assess muscle mass or visceral adiposity represents an important limitation. However, we partially addressed this by categorizing underweight into severity levels and adjusting for multiple metabolic and clinical variables that may reflect body composition differences. In addition, several factors that could influence both BMI status and gastrointestinal cancer mortality—including cancer screening and diagnostic history, tumour stage and histology, treatment‐related factors and markers of nutritional and frailty status—were unavailable in our database. Given these unmeasured factors, low BMI in our study population may in part reflect a marker of poor general health rather than a direct determinant of gastrointestinal cancer mortality, and residual confounding cannot be excluded.

In conclusion, the present study found that both underweight and obesity were significantly associated with increased gastrointestinal cancer mortality risk among individuals with T2DM, with underweight showing a substantially higher risk than obesity in a reverse J‐shaped pattern. The association between BMI and mortality risk varied across individual gastrointestinal cancer types. Further studies are warranted to explore the underlying mechanisms and establish optimal weight management guidelines in this population.

## Funding

This research was supported by a grant of the Korea Health Technology R&D Project through the Korea Health Industry Development Institute (KHIDI), funded by the Ministry of Health & Welfare, Republic of Korea (grant number HI23C0679) and by the G‐LAMP Program of the National Research Foundation of Korea (NRF) grant funded by the Ministry of Education, Republic of Korea (grant number RS‐2025‐25441317).

## Ethics Statement

This manuscript complies with the ethical guidelines for authorship and publication established by the *Journal of Cachexia, Sarcopenia and Muscle*. This study adhered to the tenets of the Declaration of Helsinki and was approved by the Kangbuk Samsung Medical Center Institutional Review Board (KBSMC 2025‐02‐019), Seoul, Korea. The Institutional Review Board waived the requirement for informed consent as de‐identified information was used for the analyses.

## Conflicts of Interest

The authors declare no conflicts of interest.

## Supporting information


**Figure S1:** Flowchart of study population derivation.
**Figure S2:** Log‐minus‐log plots for assessment of the proportional hazards assumption for BMI status in the Cox model for all gastrointestinal cancer mortality.
**Table S1:** Baseline characteristics of the study participants according to underweight and nonunderweight status.
**Table S2:** Risk of mortality from small intestine and anal cancer according to BMI status in individuals with T2DM.
**Table S3:** Incidence of gastrointestinal cancers according to BMI status in individuals with T2DM.
**Table S4:** Subgroup analysis of all gastrointestinal cancers mortality according to underweight status in individuals with T2DM.
**Table S5:** Subgroup analysis of oesophageal cancer mortality according to underweight status in individuals with T2DM.
**Table S6:** Subgroup analysis of gastric cancer mortality according to underweight status in individuals with T2DM.
**Table S7:** Subgroup analysis of colorectal cancer mortality according to underweight status in individuals with T2DM.
**Table S8:** Risk of mortality from gastrointestinal cancers according to underweight status applying competing risk analysis.
**Table S9:** Risk of mortality from gastrointestinal cancers according to underweight status applying 2‐year lag period.
**Table S10:** Risk of mortality from gastrointestinal cancers according to underweight status applying 3‐year lag period.
**Table S11:** Risk of mortality from gastrointestinal cancers according to underweight status applying 4‐year lag period.

## Data Availability

The data used in this study were obtained from the Korean NHIS database. Access to these data is available upon reasonable request and approval from the Korean NHIS. Due to privacy regulations and data sharing policies, the data cannot be made publicly available.
