# Supplementary material for: Underweight Versus Obesity and Gastrointestinal Cancer Mortality in Type 2 Diabetes: A Nationwide Cohort Study
Source: J Cachexia Sarcopenia Muscle. 2026 Oct 21;17(5):e70408. doi: 10.1002/jcsm.70408 (PMC13687580; doi:10.1002/jcsm.70408)
Supplement: Supplementary file 1 [file JCSM-17-e70408-s001.docx]

**Online supplemental materials**

**Contents**

**Supplementary Figure 1.** Flowchart of study population derivation	2

**Supplementary Figure 2.** Log-minus-log plots for assessment of the proportional hazards assumption for BMI status in the Cox model for all gastrointestinal cancer mortality	3

**Supplementary Table 1.** Baseline characteristics of the study participants according to underweight and non-underweight status	4

**Supplementary Table 2.** Risk of mortality from small intestine and anal cancer according to BMI status in individuals with T2DM	6

**Supplementary Table 3.** Incidence of gastrointestinal cancers according to BMI status in individuals with T2DM	7

**Supplementary Table 4.** Subgroup analysis of all gastrointestinal cancers mortality according to underweight status in individuals with T2DM	9

**Supplementary Table 5.** Subgroup analysis of esophageal cancer mortality according to underweight status in individuals with T2DM	11

**Supplementary Table 6.** Subgroup analysis of gastric cancer mortality according to underweight status in individuals with T2DM	13

**Supplementary Table 7.** Subgroup analysis of colorectal cancer mortality according to underweight status in individuals with T2DM	15

**Supplementary Table 8.** Risk of mortality from gastrointestinal cancers according to underweight status applying competing risk analysis	17

**Supplementary Table 9.** Risk of mortality from gastrointestinal cancers according to underweight status applying 2-year lag period	18

**Supplementary Table 10.** Risk of mortality from gastrointestinal cancers according to underweight status applying 3-year lag period	19

**Supplementary Table 11.** Risk of mortality from gastrointestinal cancers according to underweight status applying 4-year lag period	20

**Supplementary Figure 1. Flowchart of study population derivation**

T2DM, type 2 diabetes mellitus; NHIS, national health insurance service.

**Supplementary Figure 2.** **Log-minus-log plots for assessment of the proportional hazards assumption for BMI status, derived from unadjusted Kaplan-Meier curves for all gastrointestinal cancer mortality**

Log-minus-log plots for assessment of the proportional hazards assumption for BMI status (severe underweight, moderate underweight, mild underweight, and non-underweight), derived from unadjusted Kaplan-Meier curves for all gastrointestinal cancer mortality. Curves are ordered from top to bottom as severe underweight, moderate underweight, mild underweight, and non-underweight, based on their relative position at the end of follow-up. Parallel, non-crossing curves indicate that the proportional hazards assumption is reasonably satisfied. The curves remained distinctly separated during the earlier follow-up period, whereas some convergence was observed beyond approximately 2–3 years, coinciding with a marked reduction in the number of individuals remaining at risk due to progressive censoring; this should be considered when interpreting the plot at longer durations. BMI, body mass index.

**Supplementary Table 1. Baseline characteristics of the study participants according to underweight and non-underweight status**

|  | **Total** | **BMI Group** | | | | **p-value** |
| --- | --- | --- | --- | --- | --- | --- |
|  |  | **Underweight (+)** | | | **Underweight (-)** |  |
|  |  | **Severe** | **Moderate** | **Mild** |  |  |
|  |  | < 16.0 kg/m² | 16.0–16.9 kg/m² | 17.0–18.4 kg/m² | ≥ 18.5 kg/m² |  |
| Number | 2,507,976 | 2,568 | 5,444 | 26,147 | 2,473,817 |  |
| Age (years) | 59.6 ± 12.0 | 65.4 ± 15.3 | 63.4 ± 15.0 | 62.1 ± 14.2 | 59.6 ± 12.0 | <.0001 |
| < 65 | 1,643,495 (65.5) | 1,198 (46.7) | 2,830 (52.0) | 14,620 (55.9) | 1,624,847 (65.7) |  |
| ≥ 65 | 864,481 (34.5) | 1,370 (53.4) | 2,614 (48.0) | 11,527 (44.1) | 848,970 (34.3) |  |
| Sex (male) | 1,510,574 (60.2) | 1,272 (49.5) | 2,854 (52.4) | 13,986 (53.5) | 1,492,462 (60.3) | <.0001 |
| Height (cm) | 162.8 ± 9.4 | 159.5 ± 9.7 | 160.3 ± 9.4 | 160.9 ± 9.2 | 162.8 ± 9.4 | <.0001 |
| Weight (kg) | 67.4 ± 12.6 | 38.6 ± 5.1 | 42.6 ± 5.1 | 46.4 ± 5.4 | 67.7 ± 12.4 | <.0001 |
| BMI (kg/m²) | 25.3 ± 3.5 | 15.1 ± 0.8 | 16.5 ± 0.3 | 17.9 ± 0.4 | 25.4 ± 3.4 | <.0001 |
| ***Social history*** |  |  |  |  |  |  |
| Low income level (n, %) | 540,433 (21.6) | 731 (28.5) | 1,486 (27.3) | 6,759 (25.9) | 531,457 (21.5) | <.0001 |
| Regular exercise (n, %) | 543,869 (21.7) | 264 (10.3) | 817 (15.0) | 4,629 (17.7) | 538,159 (21.8) | <.0001 |
| Smoking (n, %) |  |  |  |  |  | <.0001 |
| Never smoker | 1,380,031 (55.0) | 1,560 (60.8) | 3,178 (58.4) | 15,054 (57.6) | 1,360,239 (55.0) |  |
| Ex-smoker | 563,231 (22.5) | 299 (11.6) | 710 (13.0) | 3,688 (14.1) | 558,534 (22.6) |  |
| Current smoker | 564,714 (22.5) | 709 (27.6) | 1,556 (28.6) | 7,405 (28.3) | 555,044 (22.4) |  |
| Drinking (n, %) |  |  |  |  |  | <.0001 |
| None | 1,450,549 (57.8) | 1,884 (73.4) | 3,821 (70.2) | 17,401 (66.6) | 1,427,443 (57.7) |  |
| Mild | 829,816 (33.1) | 507 (19.7) | 1252 (23.0) | 6,815 (26.1) | 821,242 (33.2) |  |
| Heavy | 227,611 (9.1) | 177 (6.9) | 371 (6.8) | 1,931 (7.39) | 225,132 (9.1) |  |
| ***Medical history*** |  |  |  |  |  |  |
| Hypertension (n, %) | 1,558,506 (62.1) | 1,134 (44.2) | 2,413 (44.3) | 11,354 (43.4) | 1,543,605 (62.4) | <.0001 |
| Dyslipidemia (n, %) | 1,458,463 (58.2) | 848 (33.0) | 1,841 (33.8) | 9,863 (37.7) | 1,445,911 (58.5) | <.0001 |
| Chronic kidney disease (n, %) | 273,902 (10.9) | 417 (16.2) | 794 (14.6) | 3,331 (12.7) | 269,360 (10.9) | <.0001 |
| Cancer other than GI cancer (n, %) | 104,918 (4.2) | 138 (5.4) | 374 (6.9) | 1572 (6.0) | 102,834 (4.2) | <.0001 |
| Diabetes duration (n, %) |  |  |  |  |  | <.0001 |
| New onset | 757,439 (30.2) | 871 (33.9) | 1,765 (32.4) | 8,113 (31.0) | 746,690 (30.2) |  |
| < 5 years | 594,245 (23.7) | 488 (19.0) | 983 (18.1) | 4,689 (17.9) | 588,085 (23.8) |  |
| < 10 years | 523,667 (20.9) | 497 (19.4) | 1,013 (18.6) | 4,932 (18.9) | 517,225 (20.9) |  |
| ≥ 10 years | 632,625 (25.2) | 712 (27.7) | 1,683 (30.9) | 8,413 (32.2) | 621,817 (25.1) |  |
| ≥ 3 oral anti-diabetic drugs (n, %) | 595,922 (23.8) | 475 (18.5) | 1,162 (21.3) | 5,847 (22.4) | 588,438 (23.8) | <.0001 |
| Insulin medication (n, %) | 213924 (8.5) | 378 (14.7) | 827 (15.2) | 3,727 (14.3) | 208,992 (8.5) | <.0001 |
| ***Metabolic profile*** |  |  |  |  |  |  |
| SBP (mmHg) | 128.5 ± 15.1 | 119.4 ± 17.1 | 120.5 ± 16.9 | 121.9 ± 16.5 | 128.6 ± 15.0 | <.0001 |
| DBP (mmHg) | 78.1 ± 10.0 | 73.64 ± 10.7 | 73.9 ± 10.6 | 74.2 ± 10.3 | 78.1 ± 9.9 | <.0001 |
| Fasting glucose (mg/dL) | 144.5 ± 45.8 | 153.9 ± 75.3 | 150.7 ± 68.4 | 149.0 ± 62.7 | 144.5 ± 45.5 | <.0001 |
| Total cholesterol (mg/dL) | 185.2 ± 43.7 | 176.2 ± 42.3 | 176.6 ± 41.6 | 177.2 ± 41.0 | 185.3 ± 43.8 | <.0001 |
| LDL-C (mg/dL) | 103.1 ± 38.4 | 94.5 ± 36.3 | 94.8 ± 35.6 | 96.1 ± 35.9 | 103.2 ± 38.4 | <.0001 |
| HDL-C (mg/dL) | 51.0 ± 14.7 | 59.7 ± 19.1 | 59.9 ± 21.5 | 58.9 ± 17.5 | 50.8 ± 14.6 | <.0001 |
| Triglyceride* (mg/dL) | 137.7 (137.6, 137.8) | 96.5 (94.6, 98.3) | 95.1 (93.7, 96.4) | 95.5 (94.9, 96.1) | 138.4 (138.3, 138.5) | <.0001 |
| eGFR (mL/min/1.73m²) | 84.1 ± 20.2 | 82.6 ± 22.7 | 83.8 ± 22.7 | 84.6 ± 21.9 | 84.1 ± 20.2 | <.0001 |
| Hemoglobin (g/dL) | 14.1 ± 1.7 | 12.7 ± 1.9 | 12.9 ± 1.9 | 13.2 ± 1.8 | 14.1 ± 1.7 | <.0001 |
| AST (U/L) | 29.9 ± 22.2 | 33.3 ± 39.0 | 32.6 ± 43.4 | 30.6 ± 37.9 | 29.9 ± 21.9 | <.0001 |
| ALT (U/L) | 31.9 ± 26.8 | 23.3 ± 29.3 | 23.5 ± 27.5 | 23.1 ± 25.9 | 32.0 ± 26.7 | <.0001 |
| r-GTP (U/L) | 53.1 ± 75.5 | 66.2 ± 142.3 | 62.0 ± 135.4 | 56.6 ± 126.3 | 53.1 ± 74.5 | <.0001 |

Data are presented as mean ± standard deviation, *geometric mean (95% confidence interval), or number (%).

BMI, body mass index; GI, gastrointestinal; SBP, systolic blood pressure; DBP, diastolic blood pressure; HDL-C, high-density lipoprotein cholesterol; LDL-C, low-density lipoprotein cholesterol; AST, aspartate aminotransferase; ALT, alanine aminotransferase; r-GTP, gamma-glutamyl transferase.

**Supplementary Table 2. Risk of mortality from small intestine and anal cancer according to BMI status in individuals with T2DM**

|  |  |  |  |  |  | **Hazard ratios (95% CI)** | | |
| --- | --- | --- | --- | --- | --- | --- | --- | --- |
|  | **BMI (kg/m²)** | **N** | **Events** | **Person-years** | **Incidence rate†** | **Model 1*** | **Model 2**** | **Model 3***** |
| **Small intestine cancer** | < 16 | 2,568 | 1 | 11,638 | 0.09 | 5.86 (0.82–41.95) | 4.02 (0.56–28.92) | 3.46 (0.48–25.00) |
|  | 16–16.9 | 5,444 | 2 | 26,902 | 0.07 | 5.03 (1.24–20.41) | 3.72 (0.91–15.11) | 3.21 (0.79–13.12) |
|  | 17–18.4 | 26,147 | 4 | 137,516 | 0.03 | 1.96 (0.72–5.32) | 1.54 (0.57–4.20) | 1.37 (0.50–3.77) |
|  | 18.5–22.9 | 586,301 | 72 | 3,321,490 | 0.02 | 1.45 (1.06–1.97) | 1.22 (0.90–1.67) | 1.18 (0.86–1.62) |
|  | 23–24.9 | 606,198 | 72 | 3,498,210 | 0.02 | 1.37 (1.01–1.87) | 1.21 (0.89–1.65) | 1.20 (0.88–1.64) |
|  | 25–29.9 | 1,045,331 | 91 | 6,063,100 | 0.02 | 1 (ref.) | 1 (ref.) | 1 (ref.) |
|  | 30–34.9 | 202,862 | 10 | 1,176,123 | 0.01 | 0.57 (0.30–1.09) | 0.80 (0.42–1.54) | 0.78 (0.41–1.51) |
|  | ≥ 35 | 33,125 | 3 | 191,023 | 0.02 | 1.05 (0.33–3.31) | 2.32 (0.73–7.37) | 2.19 (0.69–6.98) |
| **Anal cancer** | < 16 | 2,568 | 0 | 11,638 | 0.00 | N/A | N/A | N/A |
|  | 16–16.9 | 5,444 | 0 | 26,902 | 0.00 | N/A | N/A | N/A |
|  | 17–18.4 | 26,147 | 2 | 137,516 | 0.01 | 6.81 (1.54–30.19) | 4.98 (1.12–22.22) | 5.20 (1.14–23.75) |
|  | 18.5–22.9 | 586,301 | 14 | 3,321,490 | 0.00 | 1.97 (0.93–4.19) | 1.63 (0.76–3.47) | 1.74 (0.81–3.77) |
|  | 23–24.9 | 606,198 | 8 | 3,498,210 | 0.00 | 1.07 (0.44–2.57) | 0.96 (0.40–2.31) | 1.00 (0.41–2.42) |
|  | 25–29.9 | 1,045,331 | 13 | 6,063,100 | 0.00 | 1 (ref.) | 1 (ref.) | 1 (ref.) |
|  | 30–34.9 | 202,862 | 3 | 1,176,123 | 0.00 | 1.19 (0.34–4.17) | 1.51 (0.43–5.33) | 1.42 (0.40–5.01) |
|  | ≥ 35 | 33,125 | 0 | 191,023 | 0.00 | N/A | N/A | N/A |

T2DM, type 2 diabetes mellitus; 95% CI, 95% confidence interval; BMI, body mass index, †Incidence rate per 1,000 person-years.

*Model 1: Non-adjusted; **Model 2: Adjusted for Age, Sex; ***Model 3: Adjusted for factors in model 2 and Income, Smoking, Drinking, Physical activity, Hypertension, Dyslipidemia, Chronic kidney disease, Fasting glucose, ≥ 3 Oral anti-diabetic drugs, Insulin medication, and T2DM duration.

**Supplementary Table 3. Incidence of gastrointestinal cancers according to BMI status in individuals with T2DM**

|  |  |  |  |  | **Hazard ratios (95% CI)** | | |
| --- | --- | --- | --- | --- | --- | --- | --- |
|  | **BMI (kg/m²)** | **N** | **Events** | **Incidence rate†** | **Model 1*** | **Model 2**** | **Model 3***** |
| **All gastrointestinal cancers** | < 16 | 2,568 | 48 | 4.60 | 1.23 (0.93, 1.64) | 1.02 (0.77, 1.35) | 0.98 (0.74, 1.31) |
|  | 16–16.9 | 5,444 | 101 | 4.25 | 1.13 (0.93, 1.38) | 0.99 (0.81, 1.20) | 0.96 (0.79, 1.17) |
|  | 17–18.4 | 26,147 | 542 | 4.39 | 1.17 (1.07, 1.27) | 1.05 (0.97, 1.15) | 1.03 (0.95, 1.12) |
|  | 18.5–22.9 | 586,301 | 12549 | 4.09 | 1.09 (1.06, 1.11) | 0.99 (0.96, 1.01) | 0.98 (0.96, 1.00) |
|  | 23–24.9 | 606,198 | 13120 | 4.02 | 1.07 (1.04, 1.09) | 0.98 (0.95, 1.00) | 0.98 (0.96, 1.00) |
|  | 25–29.9 | 1,045,331 | 21462 | 3.76 | 1 (Ref.) | 1 (Ref.) | 1 (Ref.) |
|  | 30–34.9 | 202,862 | 3375 | 3.02 | 0.80 (0.77, 0.83) | 1.05 (1.01, 1.08) | 1.04 (1.00, 1.08) |
|  | ≥ 35 | 33,125 | 414 | 2.26 | 0.60 (0.55, 0.66) | 1.10 (0.99, 1.21) | 1.10 (0.99, 1.21) |
| **Esophageal cancer** | < 16 | 2,568 | 2 | 0.19 | 1.41 (0.35, 5.64) | 1.33 (0.33, 5.32) | 1.29 (0.32, 5.17) |
|  | 16–16.9 | 5,444 | 6 | 0.25 | 1.84 (0.82, 4.11) | 1.72 (0.77, 3.83) | 1.69 (0.75, 3.77) |
|  | 17–18.4 | 26,147 | 62 | 0.50 | 3.64 (2.81, 4.71) | 3.40 (2.62, 4.40) | 3.38 (2.60, 4.39) |
|  | 18.5–22.9 | 586,301 | 714 | 0.23 | 1.67 (1.51, 1.85) | 1.52 (1.37, 1.68) | 1.55 (1.39, 1.72) |
|  | 23–24.9 | 606,198 | 548 | 0.17 | 1.20 (1.08, 1.34) | 1.07 (0.96, 1.19) | 1.09 (0.98, 1.22) |
|  | 25–29.9 | 1,045,331 | 796 | 0.14 | 1 (Ref.) | 1 (Ref.) | 1 (Ref.) |
|  | 30–34.9 | 202,862 | 75 | 0.07 | 0.48 (0.38, 0.61) | 0.73 (0.57, 0.92) | 0.72 (0.56, 0.91) |
|  | ≥ 35 | 33,125 | 4 | 0.02 | 0.16 (0.06, 0.42) | 0.40 (0.15, 1.08) | 0.41 (0.15, 1.10) |
| **Gastric cancer** | < 16 | 2,568 | 16 | 1.53 | 0.91 (0.56, 1.49) | 0.78 (0.48, 1.28) | 0.76 (0.47, 1.25) |
|  | 16–16.9 | 5,444 | 35 | 1.47 | 0.88 (0.63, 1.22) | 0.78 (0.56, 1.09) | 0.76 (0.55, 1.06) |
|  | 17–18.4 | 26,147 | 214 | 1.72 | 1.03 (0.90, 1.18) | 0.94 (0.82, 1.08) | 0.92 (0.80, 1.05) |
|  | 18.5–22.9 | 586,301 | 5534 | 1.79 | 1.07 (1.04, 1.11) | 0.98 (0.94, 1.01) | 0.97 (0.93, 1) |
|  | 23–24.9 | 606,198 | 5799 | 1.77 | 1.05 (1.02, 1.09) | 0.96 (0.93, 0.99) | 0.96 (0.93, 0.99) |
|  | 25–29.9 | 1,045,331 | 9594 | 1.67 | 1 (Ref.) | 1 (Ref.) | 1 (Ref.) |
|  | 30–34.9 | 202,862 | 1370 | 1.22 | 0.73 (0.69, 0.77) | 0.98 (0.92, 1.03) | 0.98 (0.93, 1.04) |
|  | ≥ 35 | 33,125 | 160 | 0.87 | 0.52 (0.45, 0.61) | 1.00 (0.86, 1.18) | 1.02 (0.87, 1.19) |
| **Colorectal cancer** | < 16 | 2,568 | 31 | 2.97 | 1.38 (0.97, 1.96) | 1.11 (0.78, 1.58) | 1.06 (0.75, 1.51) |
|  | 16–16.9 | 5,444 | 64 | 2.68 | 1.24 (0.97, 1.58) | 1.07 (0.83, 1.36) | 1.03 (0.81, 1.32) |
|  | 17–18.4 | 26,147 | 309 | 2.49 | 1.15 (1.03, 1.29) | 1.02 (0.92, 1.15) | 1.00 (0.89, 1.12) |
|  | 18.5–22.9 | 586,301 | 7200 | 2.34 | 1.07 (1.04, 1.10) | 0.97 (0.94, 1.00) | 0.97 (0.94, 1.00) |
|  | 23–24.9 | 606,198 | 7672 | 2.34 | 1.07 (1.04, 1.10) | 0.98 (0.96, 1.01) | 0.99 (0.96, 1.02) |
|  | 25–29.9 | 1,045,331 | 12490 | 2.18 | 1 (Ref.) | 1 (Ref.) | 1 (Ref.) |
|  | 30–34.9 | 202,862 | 2123 | 1.89 | 0.87 (0.83, 0.91) | 1.11 (1.06, 1.16) | 1.10 (1.05, 1.15) |
|  | ≥ 35 | 33,125 | 272 | 1.48 | 0.68 (0.60, 0.77) | 1.18 (1.05, 1.33) | 1.17 (1.04, 1.32) |

Small intestine and anal cancer were not included due to the small number of incident cases, which precluded reliable risk estimation

T2DM, type 2 diabetes mellitus; 95% CI, 95% confidence interval; BMI, body mass index, †Incidence rate per 1,000 person-years.

*Model 1: Non-adjusted; **Model 2: Adjusted for Age, Sex; ***Model 3: Adjusted for factors in model 2 and Income, Smoking, Drinking, Physical activity, Hypertension, Dyslipidemia, Chronic kidney disease, Fasting glucose, ≥ 3 Oral anti-diabetic drugs, Insulin medication, and T2DM duration.

**Supplementary Table 4. Subgroup analysis of all gastrointestinal cancers mortality according to underweight status in individuals with T2DM**

| **Subgroup** | **Criteria** | **BMI (kg/m2)** | **N** | **Event** | **Duration** | **IR per 1,000** | **Adjusted HR* (95% CI)** | ***P* for interaction** |
| --- | --- | --- | --- | --- | --- | --- | --- | --- |
| **Age (years)** | Age < 65 | < 16 | 1,198 | 11 | 6,219 | 1.77 | 4.34 (2.40–7.85) | 0.0002 |
|  |  | 16–16.9 | 2,830 | 18 | 15,418 | 1.17 | 2.91 (1.83–4.62) |  |
|  |  | 17–18.4 | 14,620 | 93 | 81,944 | 1.13 | 2.95 (2.40–3.62) |  |
|  |  | ≥ 18.5 | 1,624,847 | 3,371 | 9,502,739 | 0.35 | 1 (ref.) |  |
|  | Age ≥ 65 | < 16 | 1,370 | 26 | 5,419 | 4.80 | 2.04 (1.39–3.00) |  |
|  |  | 16–16.9 | 2,614 | 48 | 11,484 | 4.18 | 1.86 (1.40–2.48) |  |
|  |  | 17–18.4 | 11,527 | 216 | 55,572 | 3.89 | 1.88 (1.64–2.15) |  |
|  |  | ≥ 18.5 | 848,970 | 7,195 | 4,747,207 | 1.52 | 1 (ref.) |  |
| **Sex** | Male | < 16 | 1,272 | 32 | 5,548 | 5.77 | 3.78 (2.67–5.36) | 0.003 |
|  |  | 16–16.9 | 2,854 | 47 | 13,616 | 3.45 | 2.38 (1.79–3.18) |  |
|  |  | 17–18.4 | 13,986 | 216 | 71,610 | 3.02 | 2.20 (1.92–2.52) |  |
|  |  | ≥ 18.5 | 1,492,462 | 7,423 | 8,559,707 | 0.87 | 1 (ref.) |  |
|  | Female | < 16 | 1,296 | 5 | 6,090 | 0.82 | 0.76 (0.32–1.82) |  |
|  |  | 16–16.9 | 2,590 | 19 | 13,286 | 1.43 | 1.61 (1.02–2.52) |  |
|  |  | 17–18.4 | 12,161 | 93 | 65,906 | 1.41 | 1.97 (1.60–2.42) |  |
|  |  | ≥ 18.5 | 981,355 | 3,143 | 5,690,239 | 0.55 | 1 (ref.) |  |
| **Cancer other than GI cancer** | No | < 16 | 2,430 | 31 | 11,064 | 2.80 | 2.35 (1.65–3.34) | 0.6254 |
|  |  | 16–16.9 | 5,070 | 53 | 25,209 | 2.10 | 1.94 (1.48–2.55) |  |
|  |  | 17–18.4 | 24,575 | 267 | 129,966 | 2.05 | 2.11 (1.87–2.39) |  |
|  |  | ≥ 18.5 | 2,370,983 | 9440 | 13,687,852 | 0.69 | 1 (ref.) |  |
|  | Yes | < 16 | 138 | 6 | 573 | 10.46 | 3.23 (1.45–7.19) |  |
|  |  | 16–16.9 | 374 | 13 | 1,693 | 7.68 | 2.73 (1.58–4.72) |  |
|  |  | 17–18.4 | 1,572 | 42 | 7,550 | 5.56 | 2.04 (1.50–2.78) |  |
|  |  | ≥ 18.5 | 102,834 | 1126 | 562,094 | 2.00 | 1 (ref.) |  |
| **CKD** | No | < 16 | 2,151 | 31 | 10,033 | 3.09 | 2.71 (1.90–3.86) | 0.0405 |
|  |  | 16–16.9 | 4,650 | 51 | 23,704 | 2.15 | 2.14 (1.63–2.82) |  |
|  |  | 17–18.4 | 22,816 | 261 | 122,641 | 2.13 | 2.30 (2.03–2.60) |  |
|  |  | ≥ 18.5 | 2,204,457 | 8,183 | 12,789,846 | 0.64 | 1 (ref.) |  |
|  | Yes | < 16 | 417 | 6 | 1,605 | 3.74 | 1.66 (0.75–3.71) |  |
|  |  | 16–16.9 | 794 | 15 | 3,198 | 4.69 | 1.94 (1.17–3.23) |  |
|  |  | 17–18.4 | 3,331 | 48 | 14,875 | 3.23 | 1.51 (1.13–2.01) |  |
|  |  | ≥ 18.5 | 269,360 | 2,383 | 1,460,100 | 1.63 | 1 (ref.) |  |
| **Smoking** | Never smoker | < 16 | 1,560 | 15 | 7,017 | 2.14 | 1.72 (1.03–2.85) | 0.1182 |
|  |  | 16–16.9 | 3,178 | 32 | 15,765 | 2.03 | 1.86 (1.32–2.64) |  |
|  |  | 17–18.4 | 15,054 | 145 | 79,766 | 1.82 | 1.89 (1.60–2.23) |  |
|  |  | ≥ 18.5 | 1,360,239 | 5,603 | 7,849,881 | 0.71 | 1 (ref.) |  |
|  | Ex-smoker | < 16 | 299 | 8 | 1,272 | 6.29 | 3.47 (1.74–6.94) |  |
|  |  | 16–16.9 | 710 | 11 | 3,360 | 3.27 | 1.93 (1.07–3.50) |  |
|  |  | 17–18.4 | 3,688 | 70 | 18,837 | 3.72 | 2.39 (1.88–3.03) |  |
|  |  | ≥ 18.5 | 558,534 | 2,858 | 3,216,937 | 0.89 | 1 (ref.) |  |
|  | Current smoker | < 16 | 709 | 14 | 3,348 | 4.18 | 3.51 (2.07–5.94) |  |
|  |  | 16–16.9 | 1,556 | 23 | 7,777 | 2.96 | 2.66 (1.76–4.02) |  |
|  |  | 17–18.4 | 7,405 | 94 | 38,913 | 2.42 | 2.39 (1.94–2.94) |  |
|  |  | ≥ 18.5 | 555,044 | 2,105 | 3,183,128 | 0.66 | 1 (ref.) |  |

T2DM, type 2 diabetes mellitus; BMI, body mass index, IR, incidence rate; HR, hazard ratio, 95% CI, 95% confidence interval; GI, gastrointestinal; CKD, chronic kidney disease

*Adjusted for age, sex, income, smoking, drinking, physical activity, hypertension, dyslipidemia, chronic kidney disease, fasting glucose, ≥ 3 oral anti-diabetic drugs, insulin use, and T2DM duration.

**Supplementary Table 5. Subgroup analysis of esophageal cancer mortality according to underweight status in individuals with T2DM**

| **Subgroup** | **Criteria** | **BMI (kg/m2)** | **N** | **Event** | **Duration** | **IR per 1,000** | **Adjusted HR* (95% CI)** | ***P* for interaction** |
| --- | --- | --- | --- | --- | --- | --- | --- | --- |
| **Age (years)** | Age < 65 | < 16 | 1,198 | 4 | 6,219 | 0.64 | 15.02 (5.60–40.31) | 0.0059 |
|  |  | 16–16.9 | 2,830 | 6 | 15,418 | 0.39 | 9.01 (4.01–20.25) |  |
|  |  | 17–18.4 | 14,620 | 19 | 81,944 | 0.23 | 5.63 (3.53–8.96) |  |
|  |  | ≥ 18.5 | 1,624,847 | 363 | 9,502,739 | 0.04 | 1 (ref.) |  |
|  | Age ≥ 65 | < 16 | 1,370 | 5 | 5,419 | 0.92 | 6.03 (2.49–14.60) |  |
|  |  | 16–16.9 | 2,614 | 6 | 11,484 | 0.52 | 3.29 (1.47–7.38) |  |
|  |  | 17–18.4 | 11,527 | 19 | 55,572 | 0.34 | 2.18 (1.38–3.46) |  |
|  |  | ≥ 18.5 | 848,970 | 553 | 4,747,207 | 0.12 | 1 (ref.) |  |
| **Sex** | Male | < 16 | 1,272 | 9 | 5,548 | 1.62 | 9.50 (4.91–18.39) | 0.9953 |
|  |  | 16–16.9 | 2,854 | 11 | 13,616 | 0.81 | 4.94 (2.72–8.99) |  |
|  |  | 17–18.4 | 13,986 | 35 | 71,610 | 0.49 | 3.15 (2.24–4.44) |  |
|  |  | ≥ 18.5 | 1,492,462 | 862 | 8,559,707 | 0.10 | 1 (ref.) |  |
|  | Female | < 16 | 1,296 | 0 | 6,090 | 0.00 | N/A |  |
|  |  | 16–16.9 | 2,590 | 1 | 13,286 | 0.08 | 4.94 (0.68–35.77) |  |
|  |  | 17–18.4 | 12,161 | 3 | 65,906 | 0.05 | 3.69 (1.15–11.80) |  |
|  |  | ≥ 18.5 | 981,355 | 54 | 5,690,239 | 0.01 | 1 (ref.) |  |
| **Cancer other than GI cancer** | No | < 16 | 2,430 | 7 | 11,064 | 0.63 | 7.52 (3.56–15.87) | 0.6545 |
|  |  | 16–16.9 | 5,070 | 9 | 25,209 | 0.36 | 4.25 (2.19–8.21) |  |
|  |  | 17–18.4 | 24,575 | 33 | 129,966 | 0.25 | 3.13 (2.20–4.45) |  |
|  |  | ≥ 18.5 | 2,370,983 | 833 | 13,687,852 | 0.06 | 1 (ref.) |  |
|  | Yes | < 16 | 138 | 2 | 573 | 3.49 | 13.76 (3.37–56.16) |  |
|  |  | 16–16.9 | 374 | 3 | 1,693 | 1.77 | 8.63 (2.72–27.33) |  |
|  |  | 17–18.4 | 1,572 | 5 | 7,550 | 0.66 | 3.36 (1.36–8.29) |  |
|  |  | ≥ 18.5 | 102,834 | 83 | 562,094 | 0.15 | 1 (ref.) |  |
| **CKD** | No | < 16 | 2,151 | 8 | 10,033 | 0.80 | 9.20 (4.56–18.53) | 0.3147 |
|  |  | 16–16.9 | 4,650 | 10 | 23,704 | 0.42 | 5.12 (2.73–9.59) |  |
|  |  | 17–18.4 | 22,816 | 36 | 122,641 | 0.29 | 3.65 (2.60–5.13) |  |
|  |  | ≥ 18.5 | 2,204,457 | 745 | 12,789,846 | 0.06 | 1 (ref.) |  |
|  | Yes | < 16 | 417 | 1 | 1,605 | 0.62 | 5.22 (0.73–37.30) |  |
|  |  | 16–16.9 | 794 | 2 | 3,198 | 0.63 | 4.24 (1.05–17.12) |  |
|  |  | 17–18.4 | 3,331 | 2 | 14,875 | 0.13 | 0.98 (0.24–3.95) |  |
|  |  | ≥ 18.5 | 269,360 | 171 | 1,460,100 | 0.12 | 1 (ref.) |  |
| **Smoking** | Never smoker | < 16 | 1,560 | 2 | 7,017 | 0.29 | 6.11 (1.52–24.62) | 0.5136 |
|  |  | 16–16.9 | 3,178 | 3 | 15,765 | 0.19 | 4.37 (1.40–13.66) |  |
|  |  | 17–18.4 | 15,054 | 8 | 79,766 | 0.10 | 2.31 (1.14–4.67) |  |
|  |  | ≥ 18.5 | 1,360,239 | 288 | 7,849,881 | 0.04 | 1 (ref.) |  |
|  | Ex-smoker | < 16 | 299 | 4 | 1,272 | 3.14 | 17.53 (6.51–47.15) |  |
|  |  | 16–16.9 | 710 | 3 | 3,360 | 0.89 | 5.17 (1.66–16.15) |  |
|  |  | 17–18.4 | 3,688 | 14 | 18,837 | 0.74 | 4.63 (2.70–7.93) |  |
|  |  | ≥ 18.5 | 558,534 | 324 | 3,216,937 | 0.10 | 1 (ref.) |  |
|  | Current smoker | < 16 | 709 | 3 | 3,348 | 0.90 | 5.88 (1.88–18.37) |  |
|  |  | 16–16.9 | 1,556 | 6 | 7,777 | 0.77 | 5.16 (2.30–11.60) |  |
|  |  | 17–18.4 | 7,405 | 16 | 38,913 | 0.41 | 2.94 (1.77–4.87) |  |
|  |  | ≥ 18.5 | 555,044 | 304 | 3,183,128 | 0.10 | 1 (ref.) |  |

T2DM, type 2 diabetes mellitus; BMI, body mass index, IR, incidence rate; HR, hazard ratio, 95% CI, 95% confidence interval; GI, gastrointestinal; CKD, chronic kidney disease

*Adjusted for age, sex, income, smoking, drinking, physical activity, hypertension, dyslipidemia, chronic kidney disease, fasting glucose, ≥ 3 oral anti-diabetic drugs, insulin use, and T2DM duration.

**Supplementary Table 6. Subgroup analysis of gastric cancer mortality according to underweight status in individuals with T2DM**

| **Subgroup** | **Criteria** | **BMI (kg/m2)** | **N** | **Event** | **Duration** | **IR per 1,000** | **Adjusted HR* (95% CI)** | ***P* for interaction** |
| --- | --- | --- | --- | --- | --- | --- | --- | --- |
| **Age (years)** | Age < 65 | < 16 | 1,198 | 4 | 6,219 | 0.64 | 3.91 (1.46–10.44) | 0.0008 |
|  |  | 16–16.9 | 2,830 | 5 | 15,418 | 0.32 | 1.99 (0.83–4.79) |  |
|  |  | 17–18.4 | 14,620 | 52 | 81,944 | 0.63 | 4.04 (3.06–5.34) |  |
|  |  | ≥ 18.5 | 1,624,847 | 1366 | 9,502,739 | 0.14 | 1 (ref.) |  |
|  | Age ≥ 65 | < 16 | 1,370 | 9 | 5,419 | 1.66 | 1.75 (0.91–3.37) |  |
|  |  | 16–16.9 | 2,614 | 21 | 11,484 | 1.83 | 2.01 (1.31–3.10) |  |
|  |  | 17–18.4 | 11,527 | 95 | 55,572 | 1.71 | 2.04 (1.66–2.50) |  |
|  |  | ≥ 18.5 | 848,970 | 2839 | 4,747,207 | 0.60 | 1 (ref.) |  |
| **Sex** | Male | < 16 | 1,272 | 12 | 5,548 | 2.16 | 3.31 (1.88–5.84) | 0.1507 |
|  |  | 16–16.9 | 2,854 | 20 | 13,616 | 1.47 | 2.37 (1.53–3.68) |  |
|  |  | 17–18.4 | 13,986 | 105 | 71,610 | 1.47 | 2.51 (2.06–3.06) |  |
|  |  | ≥ 18.5 | 1,492,462 | 3086 | 8,559,707 | 0.36 | 1 (ref.) |  |
|  | Female | < 16 | 1,296 | 1 | 6,090 | 0.16 | 0.41 (0.06–2.93) |  |
|  |  | 16–16.9 | 2,590 | 6 | 13,286 | 0.45 | 1.39 (0.62–3.10) |  |
|  |  | 17–18.4 | 12,161 | 42 | 65,906 | 0.64 | 2.45 (1.80–3.33) |  |
|  |  | ≥ 18.5 | 981,355 | 1119 | 5,690,239 | 0.20 | 1 (ref.) |  |
| **Cancer other than GI cancer** | No | < 16 | 2,430 | 11 | 11,064 | 0.99 | 2.02 (1.12–3.66) | 0.008 |
|  |  | 16–16.9 | 5,070 | 19 | 25,209 | 0.75 | 1.68 (1.07–2.64) |  |
|  |  | 17–18.4 | 24,575 | 120 | 129,966 | 0.92 | 2.28 (1.90–2.74) |  |
|  |  | ≥ 18.5 | 2,370,983 | 3851 | 13,687,852 | 0.28 | 1 (ref.) |  |
|  | Yes | < 16 | 138 | 2 | 573 | 3.49 | 3.22 (0.80–12.94) |  |
|  |  | 16–16.9 | 374 | 7 | 1,693 | 4.13 | 4.58 (2.17–9.68) |  |
|  |  | 17–18.4 | 1,572 | 27 | 7,550 | 3.58 | 4.04 (2.73–5.99) |  |
|  |  | ≥ 18.5 | 102,834 | 354 | 562,094 | 0.63 | 1 (ref.) |  |
| **CKD** | No | < 16 | 2,151 | 12 | 10,033 | 1.20 | 2.57 (1.45–4.53) | 0.0832 |
|  |  | 16–16.9 | 4,650 | 18 | 23,704 | 0.76 | 1.84 (1.16–2.93) |  |
|  |  | 17–18.4 | 22,816 | 127 | 122,641 | 1.04 | 2.72 (2.27–3.25) |  |
|  |  | ≥ 18.5 | 2,204,457 | 3302 | 12,789,846 | 0.26 | 1 (ref.) |  |
|  | Yes | < 16 | 417 | 1 | 1,605 | 0.62 | 0.73 (0.10–5.20) |  |
|  |  | 16–16.9 | 794 | 8 | 3,198 | 2.50 | 2.70 (1.34–5.41) |  |
|  |  | 17–18.4 | 3,331 | 20 | 14,875 | 1.34 | 1.63 (1.05–2.55) |  |
|  |  | ≥ 18.5 | 269,360 | 903 | 1,460,100 | 0.62 | 1 (ref.) |  |
| **Smoking** | Never smoker | < 16 | 1,560 | 5 | 7,017 | 0.71 | 1.50 (0.62–3.60) | 0.5218 |
|  |  | 16–16.9 | 3,178 | 14 | 15,765 | 0.89 | 2.13 (1.26–3.61) |  |
|  |  | 17–18.4 | 15,054 | 65 | 79,766 | 0.81 | 2.21 (1.73–2.83) |  |
|  |  | ≥ 18.5 | 1,360,239 | 2106 | 7,849,881 | 0.27 | 1 (ref.) |  |
|  | Ex-smoker | < 16 | 299 | 3 | 1,272 | 2.36 | 2.95 (0.95–9.14) |  |
|  |  | 16–16.9 | 710 | 6 | 3,360 | 1.79 | 2.41 (1.08–5.37) |  |
|  |  | 17–18.4 | 3,688 | 41 | 18,837 | 2.18 | 3.19 (2.33–4.36) |  |
|  |  | ≥ 18.5 | 558,534 | 1218 | 3,216,937 | 0.38 | 1 (ref.) |  |
|  | Current smoker | < 16 | 709 | 5 | 3,348 | 1.49 | 2.96 (1.23–7.13) |  |
|  |  | 16–16.9 | 1,556 | 6 | 7,777 | 0.77 | 1.63 (0.73–3.63) |  |
|  |  | 17–18.4 | 7,405 | 41 | 38,913 | 1.05 | 2.45 (1.79–3.36) |  |
|  |  | ≥ 18.5 | 555,044 | 881 | 3,183,128 | 0.28 | 1 (ref.) |  |

T2DM, type 2 diabetes mellitus; BMI, body mass index, IR, incidence rate; HR, hazard ratio, 95% CI, 95% confidence interval; GI, gastrointestinal; CKD, chronic kidney disease

*Adjusted for age, sex, income, smoking, drinking, physical activity, hypertension, dyslipidemia, chronic kidney disease, fasting glucose, ≥ 3 oral anti-diabetic drugs, insulin use, and T2DM duration.

**Supplementary Table 7. Subgroup analysis of colorectal cancer mortality according to underweight status in individuals with T2DM**

| **Subgroup** | **Criteria** | **BMI (kg/m2)** | **N** | **Event** | **Duration** | **IR per 1,000** | **Adjusted HR* (95% CI)** | ***P* for interaction** |
| --- | --- | --- | --- | --- | --- | --- | --- | --- |
| **Age (years)** | Age < 65 | < 16 | 1,198 | 3 | 6,219 | 0.48 | 2.57 (0.83–7.99) | 0.5344 |
|  |  | 16–16.9 | 2,830 | 7 | 15,418 | 0.45 | 2.47 (1.18–5.20) |  |
|  |  | 17–18.4 | 14,620 | 21 | 81,944 | 0.26 | 1.46 (0.95–2.25) |  |
|  |  | ≥ 18.5 | 1,624,847 | 1542 | 9,502,739 | 0.16 | 1 (ref.) |  |
|  | Age ≥ 65 | < 16 | 1,370 | 11 | 5,419 | 2.03 | 1.71 (0.94–3.09) |  |
|  |  | 16–16.9 | 2,614 | 19 | 11,484 | 1.65 | 1.48 (0.94–2.33) |  |
|  |  | 17–18.4 | 11,527 | 97 | 55,572 | 1.75 | 1.71 (1.40–2.10) |  |
|  |  | ≥ 18.5 | 848,970 | 3617 | 4,747,207 | 0.76 | 1 (ref.) |  |
| **Sex** | Male | < 16 | 1,272 | 11 | 5,548 | 1.98 | 2.99 (1.65–5.40) | 0.1991 |
|  |  | 16–16.9 | 2,854 | 14 | 13,616 | 1.03 | 1.63 (0.96–2.76) |  |
|  |  | 17–18.4 | 13,986 | 75 | 71,610 | 1.05 | 1.75 (1.39–2.20) |  |
|  |  | ≥ 18.5 | 1,492,462 | 3287 | 8,559,707 | 0.38 | 1 (ref.) |  |
|  | Female | < 16 | 1,296 | 3 | 6,090 | 0.49 | 0.78 (0.25–2.42) |  |
|  |  | 16–16.9 | 2,590 | 12 | 13,286 | 0.90 | 1.73 (0.98–3.05) |  |
|  |  | 17–18.4 | 12,161 | 43 | 65,906 | 0.65 | 1.55 (1.14–2.10) |  |
|  |  | ≥ 18.5 | 981,355 | 1872 | 5,690,239 | 0.33 | 1 (ref.) |  |
| **Cancer other than GI cancer** | No | < 16 | 2,430 | 13 | 11,064 | 1.17 | 2.00 (1.16–3.44) | 0.0481 |
|  |  | 16–16.9 | 5,070 | 23 | 25,209 | 0.91 | 1.75 (1.16–2.64) |  |
|  |  | 17–18.4 | 24,575 | 110 | 129,966 | 0.85 | 1.83 (1.51–2.21) |  |
|  |  | ≥ 18.5 | 2,370,983 | 4513 | 13,687,852 | 0.33 | 1 (ref.) |  |
|  | Yes | < 16 | 138 | 1 | 573 | 1.74 | 0.99 (0.14–6.98) |  |
|  |  | 16–16.9 | 374 | 3 | 1,693 | 1.77 | 1.12 (0.36–3.47) |  |
|  |  | 17–18.4 | 1,572 | 8 | 7,550 | 1.06 | 0.69 (0.34–1.39) |  |
|  |  | ≥ 18.5 | 102,834 | 646 | 562,094 | 1.15 | 1 (ref.) |  |
| **CKD** | No | < 16 | 2,151 | 10 | 10,033 | 1.00 | 1.79 (0.96–3.33) | 0.6298 |
|  |  | 16–16.9 | 4,650 | 21 | 23,704 | 0.89 | 1.83 (1.19–2.81) |  |
|  |  | 17–18.4 | 22,816 | 95 | 122,641 | 0.77 | 1.76 (1.43–2.16) |  |
|  |  | ≥ 18.5 | 2,204,457 | 3918 | 12,789,846 | 0.31 | 1 (ref.) |  |
|  | Yes | < 16 | 417 | 4 | 1,605 | 2.49 | 2.06 (0.77–5.50) |  |
|  |  | 16–16.9 | 794 | 5 | 3,198 | 1.56 | 1.23 (0.51–2.96) |  |
|  |  | 17–18.4 | 3,331 | 23 | 14,875 | 1.55 | 1.38 (0.91–2.09) |  |
|  |  | ≥ 18.5 | 269,360 | 1241 | 1,460,100 | 0.85 | 1 (ref.) |  |
| **Smoking** | Never smoker | < 16 | 1,560 | 7 | 7,017 | 1.00 | 1.44 (0.69–3.02) | 0.033 |
|  |  | 16–16.9 | 3,178 | 15 | 15,765 | 0.95 | 1.58 (0.95–2.62) |  |
|  |  | 17–18.4 | 15,054 | 66 | 79,766 | 0.83 | 1.58 (1.23–2.01) |  |
|  |  | ≥ 18.5 | 1,360,239 | 3061 | 7,849,881 | 0.39 | 1 (ref.) |  |
|  | Ex-smoker | < 16 | 299 | 1 | 1,272 | 0.79 | 0.98 (0.14–6.96) |  |
|  |  | 16–16.9 | 710 | 1 | 3,360 | 0.30 | 0.40 (0.06–2.85) |  |
|  |  | 17–18.4 | 3,688 | 15 | 18,837 | 0.80 | 1.17 (0.70–1.94) |  |
|  |  | ≥ 18.5 | 558,534 | 1250 | 3,216,937 | 0.39 | 1 (ref.) |  |
|  | Current smoker | < 16 | 709 | 6 | 3,348 | 1.79 | 3.66 (1.64–8.17) |  |
|  |  | 16–16.9 | 1,556 | 10 | 7,777 | 1.29 | 2.85 (1.53–5.33) |  |
|  |  | 17–18.4 | 7,405 | 37 | 38,913 | 0.95 | 2.33 (1.68–3.24) |  |
|  |  | ≥ 18.5 | 555,044 | 848 | 3,183,128 | 0.27 | 1 (ref.) |  |

T2DM, type 2 diabetes mellitus; BMI, body mass index, IR, incidence rate; HR, hazard ratio, 95% CI, 95% confidence interval; GI, gastrointestinal; CKD, chronic kidney disease

*Adjusted for age, sex, income, smoking, drinking, physical activity, hypertension, dyslipidemia, chronic kidney disease, fasting glucose, ≥ 3 oral anti-diabetic drugs, insulin use, and T2DM duration.

**Supplementary Table 8. Risk of mortality from gastrointestinal cancers according to underweight status applying competing risk analysis**

|  |  |  |  |  |  | **Hazard ratios (95% CI)** | | |
| --- | --- | --- | --- | --- | --- | --- | --- | --- |
|  | **BMI (kg/m²)** | **N** | **Events** | **Person-years** | **Incidence rate†** | **Model 1*** | **Model 2**** | **Model 3***** |
| **All gastrointestinal cancers** | Severe underweight (< 16.0) | 2,568 | 37 | 11,638 | 3.18 | 3.41 (2.47, 4.71) | 1.94 (1.40, 2.68) | 1.67 (1.21, 2.32) |
|  | Moderate underweight (16.0–16.9) | 5,444 | 66 | 26,902 | 2.45 | 2.86 (2.24, 3.64) | 1.84 (1.44, 2.35) | 1.61 (1.26, 2.06) |
|  | Mild underweight (17–18.4) | 26,147 | 309 | 137,516 | 2.25 | 2.80 (2.50, 3.13) | 2.03 (1.81, 2.27) | 1.82 (1.62, 2.04) |
|  | Non-underweight (18.5 ≤) | 2,473,817 | 10,566 | 14,249,946 | 0.74 | 1 (ref.) | 1 (ref.) | 1 (ref.) |
| **Esophageal cancer** | Severe underweight (< 16.0) | 2,568 | 9 | 11,638 | 0.77 | 9.53 (4.94, 18.37) | 6.87 (3.54, 13.33) | 5.82 (2.97, 11.39) |
|  | Moderate underweight (16.0–16.9) | 5,444 | 12 | 26,902 | 0.45 | 5.97 (3.38, 10.56) | 4.46 (2.51, 7.90) | 3.85 (2.17, 6.85) |
|  | Mild underweight (17–18.4) | 26,147 | 38 | 137,516 | 0.28 | 3.93 (2.84, 5.43) | 3.08 (2.21, 4.28) | 2.73 (1.95, 3.81) |
|  | Non-underweight (18.5 ≤) | 2,473,817 | 916 | 14,249,946 | 0.06 | 1 (ref.) | 1 (ref.) | 1 (ref.) |
| **Gastric cancer** | Severe underweight (< 16.0) | 2,568 | 13 | 11,638 | 1.12 | 3.00 (1.74, 5.17) | 1.74 (1.01, 3.02) | 1.48 (0.85, 2.56) |
|  | Moderate underweight (16.0–16.9) | 5,444 | 26 | 26,902 | 0.97 | 2.82 (1.92, 4.15) | 1.85 (1.25, 2.72) | 1.58 (1.07, 2.34) |
|  | Mild underweight (17–18.4) | 26,147 | 147 | 137,516 | 1.07 | 3.34 (2.83, 3.94) | 2.44 (2.06, 2.88) | 2.16 (1.82, 2.55) |
|  | Non-underweight (18.5 ≤) | 2,473,817 | 4,205 | 14,249,946 | 0.30 | 1 (ref.) | 1 (ref.) | 1 (ref.) |
| **Colorectal cancer** | Severe underweight (< 16.0) | 2,568 | 14 | 11,638 | 1.20 | 2.62 (1.55, 4.44) | 1.39 (0.82, 2.36) | 1.23 (0.73, 2.09) |
|  | Moderate underweight (16.0–16.9) | 5,444 | 26 | 26,902 | 0.97 | 2.30 (1.56, 3.38) | 1.42 (0.96, 2.09) | 1.26 (0.86, 1.86) |
|  | Mild underweight (17–18.4) | 26,147 | 118 | 137,516 | 0.86 | 2.18 (1.82, 2.62) | 1.54 (1.28, 1.85) | 1.41 (1.17, 1.70) |
|  | Non-underweight (18.5 ≤) | 2,473,817 | 5,159 | 14,249,946 | 0.36 | 1 (ref.) | 1 (ref.) | 1 (ref.) |

All deaths other than gastrointestinal cancer death were treated as competing events. 95% CI, 95% confidence interval; BMI, body mass index, †Incidence rate per 1,000 person-years. *Model 1: Non-adjusted; **Model 2: Adjusted for Age, Sex; ***Model 3: Adjusted for factors in model 2 and Income, Smoking, Drinking, Physical activity, Hypertension, Dyslipidemia, Chronic kidney disease, Fasting glucose, ≥ 3 Oral anti-diabetic drugs, Insulin medication, and T2DM duration.

**Supplementary Table 9. Risk of mortality from gastrointestinal cancers according to underweight status applying 2-year lag period**

|  |  |  |  |  |  | **Hazard ratios (95% CI)** | | |
| --- | --- | --- | --- | --- | --- | --- | --- | --- |
|  | **BMI (kg/m²)** | **N** | **Events** | **Person-years** | **Incidence rate†** | **Model 1*** | **Model 2**** | **Model 3***** |
| **All gastrointestinal cancers** | Severe underweight (< 16.0) | 2,314 | 26 | 9,204 | 2.82 | 3.75 (2.55–5.50) | 2.61 (1.77–3.83) | 2.24 (1.52–3.30) |
|  | Moderate underweight (16.0–16.9) | 5,101 | 49 | 21,624 | 2.27 | 2.98 (2.25–3.94) | 2.23 (1.68–2.94) | 1.96 (1.48–2.60) |
|  | Mild underweight (17–18.4) | 25,044 | 235 | 111,926 | 2.10 | 2.75 (2.42–3.13) | 2.19 (1.93–2.50) | 1.99 (1.74–2.26) |
|  | Non-underweight (18.5 ≤) | 2,451,284 | 9,030 | 11,787,034 | 0.77 | 1 (ref.) | 1 (ref.) | 1 (ref.) |
| **Esophageal cancer** | Severe underweight (< 16.0) | 2,314 | 6 | 9,204 | 0.65 | 10.08 (4.52–22.50) | 8.88 (3.97–19.84) | 7.31 (3.26–16.40) |
|  | Moderate underweight (16.0–16.9) | 5,101 | 8 | 21,624 | 0.37 | 5.68 (2.83–11.40) | 4.90 (2.44–9.84) | 4.16 (2.07–8.38) |
|  | Mild underweight (17–18.4) | 25,044 | 30 | 111,926 | 0.27 | 4.10 (2.85–5.90) | 3.54 (2.45–5.10) | 3.10 (2.14–4.49) |
|  | Non-underweight (18.5 ≤) | 2,451,284 | 775 | 11,787,034 | 0.07 | 1 (ref.) | 1 (ref.) | 1 (ref.) |
| **Gastric cancer** | Severe underweight (< 16.0) | 2,314 | 8 | 9,204 | 0.87 | 2.94 (1.47–5.88) | 2.11 (1.05–4.22) | 1.78 (0.89–3.56) |
|  | Moderate underweight (16.0–16.9) | 5,101 | 23 | 21,624 | 1.06 | 3.59 (2.39–5.42) | 2.72 (1.81–4.10) | 2.35 (1.56–3.53) |
|  | Mild underweight (17–18.4) | 25,044 | 102 | 111,926 | 0.91 | 3.07 (2.52–3.74) | 2.46 (2.02–3.00) | 2.18 (1.79–2.66) |
|  | Non-underweight (18.5 ≤) | 2,451,284 | 3,507 | 11,787,034 | 0.30 | 1 (ref.) | 1 (ref.) | 1 (ref.) |
| **Colorectal cancer** | Severe underweight (< 16.0) | 2,314 | 11 | 9,204 | 1.20 | 3.16 (1.75–5.71) | 2.08 (1.15–3.76) | 1.84 (1.02–3.32) |
|  | Moderate underweight (16.0–16.9) | 5,101 | 17 | 21,624 | 0.79 | 2.07 (1.29–3.33) | 1.49 (0.92–2.40) | 1.34 (0.83–2.16) |
|  | Mild underweight (17–18.4) | 25,044 | 97 | 111,926 | 0.87 | 2.27 (1.86–2.78) | 1.77 (1.45–2.17) | 1.64 (1.34–2.01) |
|  | Non-underweight (18.5 ≤) | 2,451,284 | 4,512 | 11,787,034 | 0.38 | 1 (ref.) | 1 (ref.) | 1 (ref.) |

95% CI, 95% confidence interval; BMI, body mass index, †Incidence rate per 1,000 person-years.

*Model 1: Non-adjusted; **Model 2: Adjusted for Age, Sex; ***Model 3: Adjusted for factors in model 2 and Income, Smoking, Drinking, Physical activity, Hypertension, Dyslipidemia, Chronic kidney disease, Fasting glucose, ≥ 3 Oral anti-diabetic drugs, Insulin medication, and T2DM duration.

**Supplementary Table 10. Risk of mortality from gastrointestinal cancers according to underweight status applying 3-year lag period**

|  |  |  |  |  |  | **Hazard ratios (95% CI)** | | |
| --- | --- | --- | --- | --- | --- | --- | --- | --- |
|  | **BMI (kg/m²)** | **N** | **Events** | **Person-years** | **Incidence rate†** | **Model 1*** | **Model 2**** | **Model 3***** |
| **All gastrointestinal cancers** | Severe underweight (< 16.0) | 2,115 | 23 | 7,034 | 3.27 | 4.18 (2.77, 6.29) | 3.04 (2.02, 4.58) | 2.64 (1.75, 3.97) |
|  | Moderate underweight (16.0–16.9) | 4,794 | 37 | 16,727 | 2.21 | 2.82 (2.04, 3.89) | 2.18 (1.58, 3.01) | 1.93 (1.40, 2.67) |
|  | Mild underweight (17–18.4) | 24,054 | 171 | 87,582 | 1.95 | 2.48 (2.13, 2.89) | 2.02 (1.73, 2.35) | 1.83 (1.57, 2.14) |
|  | Non-underweight (18.5 ≤) | 2,429,461 | 7,388 | 9,364,976 | 0.79 | 1 (Ref.) | 1 (Ref.) | 1 (Ref.) |
| **Esophageal cancer** | Severe underweight (< 16.0) | 2,115 | 6 | 7,034 | 0.85 | 12.65 (5.66, 28.26) | 11.80 (5.28, 26.39) | 9.81 (4.37, 22.02) |
|  | Moderate underweight (16.0–16.9) | 4,794 | 7 | 16,727 | 0.42 | 6.18 (2.93, 13.01) | 5.55 (2.63, 11.69) | 4.71 (2.23, 9.96) |
|  | Mild underweight (17–18.4) | 24,054 | 24 | 87,582 | 0.27 | 4.03 (2.68, 6.06) | 3.57 (2.37, 5.37) | 3.14 (2.08, 4.74) |
|  | Non-underweight (18.5 ≤) | 2,429,461 | 639 | 9,364,976 | 0.07 | 1 (Ref.) | 1 (Ref.) | 1 (Ref.) |
| **Gastric cancer** | Severe underweight (< 16.0) | 2,115 | 7 | 7,034 | 1.00 | 3.35 (1.60, 7.04) | 2.51 (1.19, 5.26) | 2.14 (1.02, 4.50) |
|  | Moderate underweight (16.0–16.9) | 4,794 | 18 | 16,727 | 1.08 | 3.62 (2.28, 5.75) | 2.83 (1.78, 4.51) | 2.47 (1.55, 3.93) |
|  | Mild underweight (17–18.4) | 24,054 | 70 | 87,582 | 0.80 | 2.68 (2.11, 3.39) | 2.19 (1.73, 2.78) | 1.96 (1.54, 2.48) |
|  | Non-underweight (18.5 ≤) | 2,429,461 | 2,804 | 9,364,976 | 0.30 | 1 (Ref.) | 1 (Ref.) | 1 (Ref.) |
| **Colorectal cancer** | Severe underweight (< 16.0) | 2,115 | 9 | 7,034 | 1.28 | 3.23 (1.68, 6.21) | 2.21 (1.15, 4.24) | 1.95 (1.02, 3.75) |
|  | Moderate underweight (16.0–16.9) | 4,794 | 12 | 16,727 | 0.72 | 1.80 (1.02, 3.18) | 1.34 (0.76, 2.36) | 1.21 (0.69, 2.14) |
|  | Mild underweight (17–18.4) | 24,054 | 71 | 87,582 | 0.81 | 2.03 (1.61, 2.57) | 1.61 (1.28, 2.04) | 1.50 (1.18, 1.90) |
|  | Non-underweight (18.5 ≤) | 2,429,461 | 3,740 | 9,364,976 | 0.40 | 1 (Ref.) | 1 (Ref.) | 1 (Ref.) |

95% CI, 95% confidence interval; BMI, body mass index, †Incidence rate per 1,000 person-years.

*Model 1: Non-adjusted; **Model 2: Adjusted for Age, Sex; ***Model 3: Adjusted for factors in model 2 and Income, Smoking, Drinking, Physical activity, Hypertension, Dyslipidemia, Chronic kidney disease, Fasting glucose, ≥ 3 Oral anti-diabetic drugs, Insulin medication, and T2DM duration.

**Supplementary Table 11. Risk of mortality from gastrointestinal cancers according to underweight status applying 4-year lag period**

|  |  |  |  |  |  | **Hazard ratios (95% CI)** | | |
| --- | --- | --- | --- | --- | --- | --- | --- | --- |
|  | **BMI (kg/m²)** | **N** | **Events** | **Person-years** | **Incidence rate†** | **Model 1*** | **Model 2**** | **Model 3***** |
| **All gastrointestinal cancers** | Severe underweight (< 16.0) | 1,948 | 18 | 5,009 | 3.59 | 4.43 (2.79, 7.04) | 3.36 (2.11, 5.33) | 2.92 (1.84, 4.64) |
|  | Moderate underweight (16.0–16.9) | 4,497 | 26 | 12,084 | 2.15 | 2.65 (1.80, 3.89) | 2.10 (1.43, 3.09) | 1.88 (1.28, 2.77) |
|  | Mild underweight (17–18.4) | 23,044 | 110 | 64,023 | 1.72 | 2.11 (1.75, 2.55) | 1.74 (1.44, 2.10) | 1.60 (1.32, 1.93) |
|  | Non-underweight (18.5 ≤) | 2,401,442 | 5,671 | 6,949,335 | 0.82 | 1 (Ref.) | 1 (Ref.) | 1 (Ref.) |
| **Esophageal cancer** | Severe underweight (< 16.0) | 1,948 | 3 | 5,009 | 0.60 | 8.27 (2.66, 25.74) | 7.98 (2.56, 24.81) | 6.78 (2.17, 21.15) |
|  | Moderate underweight (16.0–16.9) | 4,497 | 5 | 12,084 | 0.41 | 5.70 (2.36, 13.76) | 5.21 (2.16, 12.57) | 4.52 (1.86, 10.95) |
|  | Mild underweight (17–18.4) | 23,044 | 17 | 64,023 | 0.27 | 3.65 (2.25, 5.92) | 3.27 (2.01, 5.30) | 2.93 (1.80, 4.78) |
|  | Non-underweight (18.5 ≤) | 2,401,442 | 506 | 6,949,335 | 0.07 | 1 (Ref.) | 1 (Ref.) | 1 (Ref.) |
| **Gastric cancer** | Severe underweight (< 16.0) | 1,948 | 7 | 5,009 | 1.40 | 4.56 (2.17, 9.58) | 3.55 (1.69, 7.45) | 3.05 (1.45, 6.40) |
|  | Moderate underweight (16.0–16.9) | 4,497 | 12 | 12,084 | 0.99 | 3.23 (1.83, 5.70) | 2.60 (1.47, 4.58) | 2.29 (1.30, 4.04) |
|  | Mild underweight (17–18.4) | 23,044 | 46 | 64,023 | 0.72 | 2.33 (1.74, 3.12) | 1.94 (1.45, 2.59) | 1.75 (1.30, 2.34) |
|  | Non-underweight (18.5 ≤) | 2,401,442 | 2,145 | 6,949,335 | 0.31 | 1 (Ref.) | 1 (Ref.) | 1 (Ref.) |
| **Colorectal cancer** | Severe underweight (< 16.0) | 1,948 | 7 | 5,009 | 1.40 | 3.42 (1.63, 7.18) | 2.41 (1.15, 5.07) | 2.14 (1.02, 4.50) |
|  | Moderate underweight (16.0–16.9) | 4,497 | 9 | 12,084 | 0.74 | 1.81 (0.94, 3.49) | 1.39 (0.72, 2.67) | 1.26 (0.66, 2.43) |
|  | Mild underweight (17–18.4) | 23,044 | 42 | 64,023 | 0.66 | 1.60 (1.18, 2.16) | 1.29 (0.95, 1.75) | 1.20 (0.88, 1.63) |
|  | Non-underweight (18.5 ≤) | 2,401,442 | 2,860 | 6,949,335 | 0.41 | 1 (Ref.) | 1 (Ref.) | 1 (Ref.) |

95% CI, 95% confidence interval; BMI, body mass index, †Incidence rate per 1,000 person-years.

*Model 1: Non-adjusted; **Model 2: Adjusted for Age, Sex; ***Model 3: Adjusted for factors in model 2 and Income, Smoking, Drinking, Physical activity, Hypertension, Dyslipidemia, Chronic kidney disease, Fasting glucose, ≥ 3 Oral anti-diabetic drugs, Insulin medication, and T2DM duration.
